# Genomic epidemiology and population structure of *Neisseria gonorrhoeae* in Norway, 2016–2017

**DOI:** 10.1099/mgen.0.000359

**Published:** 2020-03-26

**Authors:** Kristian Alfsnes, Vegard Eldholm, Anne Olaug Olsen, Ola Brønstad Brynildsrud, Jon Bohlin, Martin Steinbakk, Dominique A. Caugant

**Affiliations:** ^1^​ Division for Infection Control and Environmental Health, Norwegian Institute of Public Health, Oslo, Norway; ^2^​ National Advisory Unit for Sexually Transmitted Infections, Oslo, Norway; ^3^​ Institute for Health and Society, Faculty of Medicine, University of Oslo, Oslo, Norway; ^4^​ Østfold Hospital Trust, Center for Laboratory Medicine, Grålum, Norway

**Keywords:** genomic epidemiology, population structure, outbreak, antimicrobial resistance

## Abstract

This study presents the nationwide epidemiology of *Neisseria gonorrhoeae *, using whole-genome sequencing of all culture-positive cases, which comprise roughly 40 % of all cases of gonorrhea reported in Norway from 2016 to 2017. Isolates were assigned to sequence types and Bayesian analysis clusters and variation in genes coding for antibiotic resistance was linked to phenotypic resistance data. The study also included isolates taken from the same patients from different anatomical sites at one or more time points. Comparing these isolates allows for observation of patterns of infections, i.e. multiple reinfections of genetically related clones vs. reinfections of genetically distant clones, and quantification of the genomic variation of closely related isolates from samples taken from a patient within the same day. Demographically, the patients in the study could be split into two groups; one group of patients from the capital with a high proportion of men who have sex with men (MSM), and another consisting of young adults with transmission primarily between males and females from outside the capital. Some clusters of *N. gonorrhoeae* were restricted to one of these two demographic groups. Pairwise comparison of multiple isolates from the same patients revealed that most were reinfected with different clones. Observations of frequent reinfections in patients is a concern and should be taken into account in the development of improved information and treatment guidelines.

## Data Summary

Sequence data have been deposited in the European Nucleotide Archive (ENA) under accession numbers PRJEB32435. All supporting data, code and protocols have been provided within the article or in the Supplementary Material.

**Impact Statement**

Gonorrhea is back with a vengeance across Europe. This paper presents the results of epidemiological surveillance of gonorrhea in Norway in 2016–2017. We use whole-genome sequencing to identify clusters in concordance with multilocus sequence types (MLST), which is widely used to compare and contrast gonorrhea outbreaks and resistance development in different countries. Findings in this study offer supporting insight into the evolution and transmission of *N. gonorrhoeae* – which may in turn enable better models and monitoring of gonorrhea for improved public health initiatives. The study will be of interest to the ongoing public health surveillance of *N. gonorrhoeae *, specifically the observations of phenotypic antibiotic resistance, which is of growing concern, as well as the epidemiology, and understanding of the transmission patterns of this pathogen.

## Introduction

*Neisseria gonorrhoeae *, is an obligate human pathogen, usually colonizing the genital mucosa, and the causative agent of gonorrhea, one of the most prevalent sexually transmitted infections worldwide, with more than 80 million cases reported annually [1, 2]. *N. gonorrhoeae* can also colonize the ocular mucosa, and is frequently detected in the oropharynx and the anal mucosa in men who have sex with men (MSM) [3]. Female genital infections as well as oropharyngeal and anal/rectal infections are less likely to be symptomatic and may go unnoticed [4]. Most infections are local and readily cured with antibiotic treatment, but they can spread and cause pelvic inflammatory disease in women and epididymo-orchitis in men [5]. Transmission from mother to child during birth can result in neonatal blindness if untreated [6]. A rare form of the disease, disseminated gonococcal infection, may result in infectious arthritis and endocarditis [7].

As an effective vaccine has yet to be developed, antibiotics represent the only effective method to treat the disease. Surveillance, screening and disease prevention (safe sex and condom use) have been instrumental in controlling the disease. In recent years, however, the bacterium has acquired resistance to most available antibiotics, rendering untreatable gonorrhea infections a definite threat [8]. Dual therapy with ceftriaxone and azithromycin is the currently recommended treatment in many countries [9]. Worryingly, strains harbouring resistance to both these drugs have been reported [10, 11]. Consequently, the World Health Organization recognized *N. gonorrhoeae* as a high-priority pathogen for which development of new antimicrobials is necessary [12]. To control the spread of antibiotic resistant gonococci, additional measures such as improved diagnosis and surveillance, as well as rapid identification of transmission links between individuals and populations are important.

The incidence of gonorrhea has increased drastically in many western countries in recent years, especially among MSM. In the European Union in 2014, 44 % of gonorrhea cases occurred among MSM [13]. In the USA, gonorrhea diagnoses increased by 67% overall over a 5 year period from 2013 to 2017, yielding a rate of 171.9 cases per 100 000 population in 2017 [14]. The number of diagnosed cases in England rose to 44 676 in 2017, a 22 % increase from the previous year [15]. In Norway, the incidence of the disease has been increasing dramatically in the past decade, resulting in an epidemic situation driven mainly by high rates of transmission among MSM. While in 1999 there were only 190 reported cases of gonorrhea (incidence 4.27 per 100 000), there were 1658 in 2018 (incidence 31.53 per 100 000); 63 % of them in the capital. Sixty-one percent of the gonococcal infections in Norway in 2018 were among MSM (https://www.fhi.no/nyheter/2019/bekymringsfull-utvikling-av-gonore-og-syfilis/). Gonorrhea is a notifiable disease in Norway, positive isolates are sent to Norwegian Institute of Public Health (NIPH) for further characterization.

Whole-genome-sequence (WGS) analyses of gonococcal isolates have provided new insights into the population structure, evolution and spread of the bacterium in different parts of the world [16–18]. WGS has also been used to elucidate the mechanisms of antimicrobial resistance in *N. gonorrhoeae* and might be an important tool to predict susceptibility, identify new resistance mechanisms and eventually it might help optimizing treatment to slow the spread of antibiotic resistance [19]. WGS allows for comparison of genetic changes occurring in the isolates within one host. Previous studies have illustrated how within-host evolution and mutation is shaping the genome, especially in phase variable loci, and resulting in antigenic variation in the closely related species *Neisseria meningitidis* [20, 21] and *Neisseria lactamica* [22].

In the present study, we report the WGS analysis of all gonococcal isolates referred to the Norwegian reference laboratory for gonococci for the years 2016–2017, providing a detailed description of the epidemiological situation. Multiple isolates recovered from individual patients, either from the same anatomical site over time or from different anatomical sites, are also described.

## Methods

### Isolate collection

The Norwegian Institute of Public Health (NIPH) was designated as the national reference laboratory for gonococci in late 2015 and received cultures of *N. gonorrhoeae* from all the Medical Microbiology laboratories in Norway from January 2016. From the 2495 cases of gonococcal infections notified to the Norwegian Surveillance System for Communicable Diseases (MSIS, http://www.msis.no/) in 2016 and 2017, 1071 isolates were received for confirmation, antimicrobial resistance testing and molecular typing. WGS data from 958 isolates were available for analysis (Table 1). More than one isolate was available from 75 out of the 873 patients in the dataset, ten of these patients represented by three isolates, the rest by two isolates (Table 1). Patient sex, age and place of residency (by county), as well as the anatomical sample site were obtained (Table 1).

**Table 1.** Isolates and demographics

No. of sequenced isolates	958
**No. of patients**	873
**No. of patients with multiple isolates**	75 (8.6 %)
Two isolates	65 (7.4 %)
Three isolates	10 (1.1 %)
Time interval (median) between isolates*	2.5 (0–443) days
**Sex (male)**	711 (81.4 %)
Age* (median)	31 (16–83) years
**Patient residency**	
Greater Oslo region†	584 (66.9 %)
Rest of Norway	350 (40.1 %)
Not defined/missing data	24 (2.7 %)
**Anatomical sample site**	
Urethra	457 (47.7 %)
Anus	207 (21.6 %)
Cervix, uterus or vagina	125 (13 %)
Throat	86 (9 %)
Other‡	18 (1.9 %)
Not defined/missing data	65 (6.8 %)

*Median and range.

†Including Oslo and Akershus counties.

‡Including a few cases isolated from ureter, fluid from knee, eye and unspecified genitalia.

Isolates were grown overnight at 37 °C in an atmosphere of 5 % CO_2_ on chocolate blood agar for DNA extraction. Antibiotic susceptibility testing (AST) was done on a GC agar base supplemented with 1 % IsoVitaleX and 1 % haemoglobin. The MIC of antibiotics were determined using E-test according to the manufacturer’s instructions (bioMérieux, Marcy-l'Étoile, France). The antibiotics tested were ciprofloxacin (CIP), ceftriaxone (CRO), cefixime (CFM), azithromycin (AZM), penicillin G (PCN), spectinomycin (SPX) and tetracycline (TET). Interpretation of results [susceptible/intermediate/resistant (SIR)] was according to clinical breakpoints set by the European Committee for Antimicrobial Susceptibility Testing (EUCAST) for the years 2016 and 2017, respectively.

### Whole-genome sequencing

DNA was extracted using MagNA Pure 96 (Roche Life Science), and DNA sequencing libraries were prepared from the extracted DNA using KAPA HyperPlus kits (Roche Life Science) with NEXTflex DNA barcodes (Bioo Scientific) following the manufacturer’s instructions. The DNA libraries were sequenced on the MiSeq sequencing platform (Illumina) using the v2 500-cycles or the v3 600-cycles reagent kits (Illumina) following the manufacturer’s instructions.

### Mapping and variant calling, phylogenetic reconstruction, typing and identification of differences

Sequence reads were trimmed and adapters removed using Trimmomatic v0.36 (adjusted settings as follows: *leading/trailing* 3; *sliding window* 3 : 15; *minimum length* 36) [23]. FASTA contigs were obtained from the trimmed reads using Spades v3.12.0 (with the *careful* option enabled) [24], and further filtered using an in-house script (removing contigs <500 nt and with <2 k-mer coverage as reported by SPAdes). Core SNPs were called from the filtered contigs using Parsnp v1.2.0 [25] with FA1090 (NC_002946.2) as reference. Locally collinear blocks (LCB) were extracted and their relative positions retained, using the reference sequence FA1090 as filler, using Harvesttools v1.2.0 [25] to produce a list of SNPs. This was subsequently used to produce a FASTA alignment using the variant call format (VCF) using BCFtools v1.9.0 and vcf-consensus from the VCFtools v0.1.16 package [26]. Recombination events were identified using Gubbins v2.34 [27], and masked from the FASTA alignment using maskrc-svg.py v0.4 (https://github.com/kwongj/maskrc-svg). The number of substitutions (SNPs) were calculated using snp-dists v0.6.2 (https://github.com/tseemann/snp-dists). Phandango [28] was used for visualization of the maximum-likelihood phylogenetic tree obtained from Gubbins v2.34 (using RAxML v8.2.12 [29]). Genome-based antimicrobial resistance (AMR) predictions were obtained from Pathogenwatch (v2.1, Wellcome Sanger Institute) using the integrated PAARSNP protocol and the included resistance element database collection (assessed August 2019). Briefly, FASTA files were searched against a library of known mutations and genes implicated in AMR in *N. gonorrhoeae* using PAARSNP (v0.0.1), developed by Simon Harris and the EuroGASP consortium, using blastn. Matches in the query with sufficient library sequence coverage and identity (defined by curator at 95 and 80 %, respectively) were assigned as ‘intermediate’ or ‘resistant’ following the curated definitions. CRO, CFM and SPX are only represented with one EUCAST breakpoint, separating ‘susceptible’ and ‘resistant’, whereas the library contained mutations and genes also encoding ‘intermediate’ level of resistance. Predicted ‘intermediate susceptibility’ genotypes for these three antimicrobials were designated as ‘susceptible’ in order to allow for comparison between the observed and predicted AMR. Observed and predicted cases of ‘intermediate susceptibility’ for all seven antibiotics were treated as ‘susceptible’ in order to calculate the sensitivity and specificity.

Multilocus sequence types (MLST) according to the PubMLST scheme (www.pubmlst.org/neisseria) were obtained using mlst v2.15 (https://github.com/tseemann/mlst) on the filtered FASTA contigs described above. The contigs were also used for *Neisseria gonorrhoeae* multi-antigen sequence typing (NG-MAST) [30] using ng-master v0.5.5 (https://github.com/MDU-PHL/ngmaster). Bayesian analysis of population structure (BAPS) clustering was determined using the *rhierBAPS* v1.1.2 package (at default settings; *max.depth=2, n.pop=number of isolates/5*) in R [31]. Genome-based clustering was also performed using Population Partitioning Using Nucleotide *K*-mers (PopPUNK) v1.1.6 (at default setting) [32].

R v3.6.1 was used to produce figures, and perform the statistical analyses.

## Results

### Epidemiology

All culture-positive *N. gonorrhoeae* isolates, approximately 40 % of all cases (MSIS, http://www.msis.no/) occurring in 2016 and 2017 are included in this study. Some isolates (*n*=123) received by the reference laboratory were omitted either due to insufficient resources (*n*=52), failure to obtain viable colonies or to extract DNA (*n*=44), or poor sequencing results (*n*=27). The failed isolates were evenly distributed in the dataset.

The dataset included 958 isolates from 873 patients (Table 1). Demographically, the majority of all patients were young males residing in the urban region encompassing greater Oslo (Table 1). The majority of the isolates were taken from the urethra (52 %), and few isolates from the cervix, uterus or vagina (14 %) – reflecting the low percentage of females among the patients (Table 1). A majority of the cases reported likely infection within the country (74 %) (MSIS, http://www.msis.no/).

Phenotypic characterization of antibiotic resistance (shown as SIR%) of isolates is presented in Table 1. All isolates were susceptible to ceftriaxone (CRO) and spectinomycin (SPX), and only ten isolates were resistant to cefixime (CFM) (Table 2). As described in methods, predicted ‘intermediate susceptibility’ resistance for these three antimicrobials was treated as ‘susceptible’ in order to compare to the single EUCAST breakpoint. The genome-based AMR prediction matched all the phenotypic observations for CRO and SPX; no prediction of resistance was made (Table 2, Fig. S1, available in the online version of this article). Genome-based predictions for ciprofloxacin (CIP) and CFM resistance matched the observations (>95 % match); however, a few false predictions reduced the sensitivity and specificity of the latter (Table 2 and Fig. S1). The phenotypic observations of the remaining three antimicrobials, azithromycin (AZM), penicillin G (PCN), tetracycline (TET), could not be accurately predicted (<66 % match, Table 2, Fig. S1). Genome-based AZM, PCN and TET predictions resulted in some false ‘susceptible’ predictions reducing sensitivity, whereas for PCN false ‘resistant’ predictions reduced the specificity (Table 2).

**Table 2.** Observed antibiotic resistance and genome-based antimicrobial resistance (AMR) prediction

	Phenotypic resistance			AMR prediction*			Match	Sensitivity†	Specificity†
	**S**	**I**	**R**	**S**	**I**	**R**			
Ciprofloxacin (CIP)	57 %	0 %	43 %	58 %	0 %	42 %	99 %	99 %	100 %
Ceftriaxone (CRO)	100 %	0 %	0 %	62 %	38 %	0 %	100 %	na	100 %
Cefixime (CFM)	99 %	0 %	1 %	62 %	32 %	6 %	95 %	83 %	95 %
Azithromycin (AZM)	58 %	32 %	10 %	66 %	31 %	3 %	66%	58 %	100 %
Penicillin G (PCN)	5 %	65 %	30 %	37 %	42 %	22 %	54 %	74 %	96 %
Spectinomycin (SPX)	100 %	0 %	0 %	100 %	0 %	0 %	100 %	na	100 %
Tetracycline (TET)	48 %	19 %	33 %	12 %	67 %	22 %	52 %	74 %	100 %

*AMR prediction by Pathogenwatch.

†Intermediate susceptible observations and predictions were treated as susceptible in order to calculate sensitivity and specificity.

SIR, S, susceptible; I, intermediate susceptibility; R, resistant.

### Genomic epidemiology

**Fig. 1.**
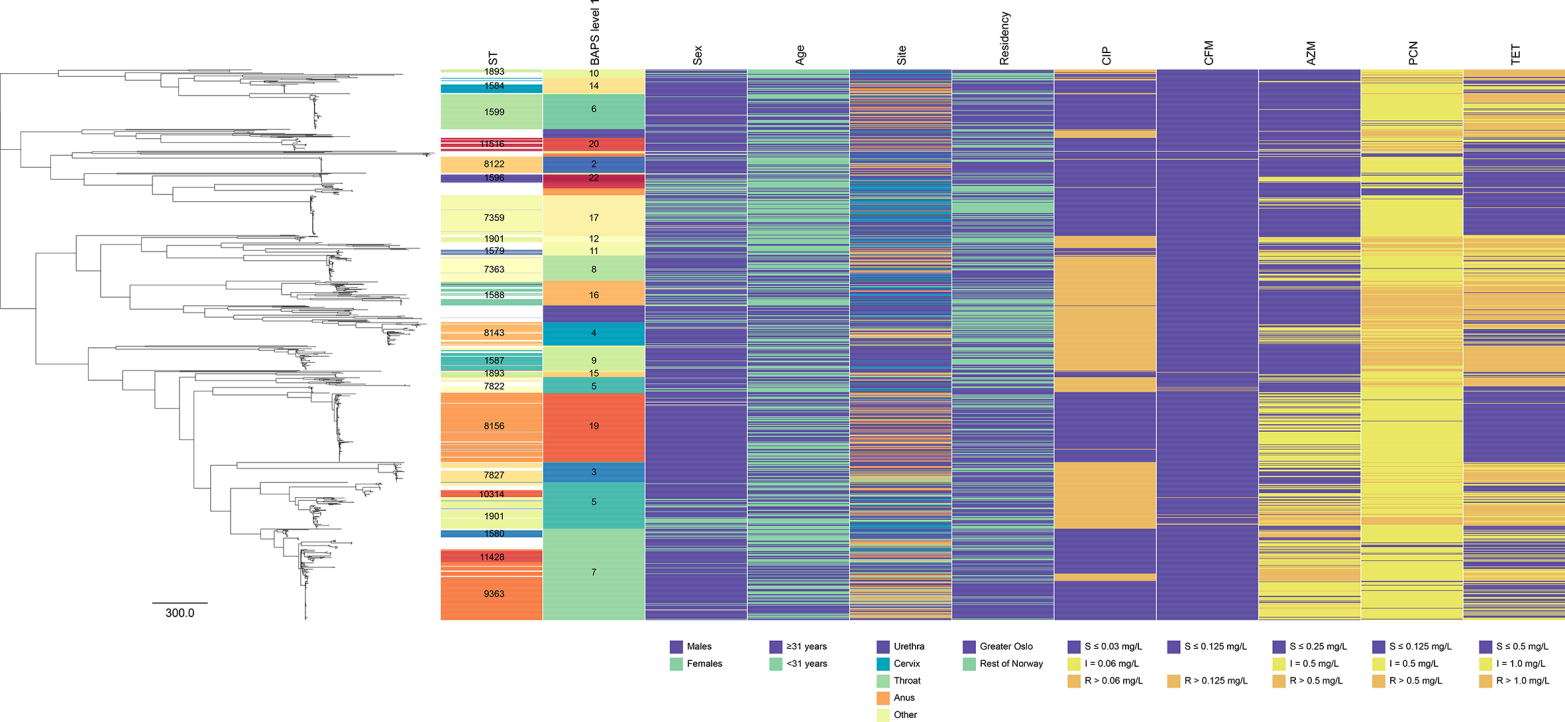
Maximum-likelihood phylogeny of all available Norwegian *N. gonorrhoeae* in the data set based on core genome SNPs. Showing STs (represented by ≥10 isolates), clusters defined by BAPS level 1, and patient data (sex, age, sampling site and residency). Phenotypic susceptible/intermediate/resistant (SIR) data using the EUCAST breakpoints shown for ciprofloxacin (CIP), cefixime (CFM), azithromycin (AZM); penicillin G (PCN), and tetracycline (TET). No isolates were observed resistant to ceftriaxone (CRO) (>0.125 mg l^−1^) or spectinomycin (SPX) (>64 mg l^−1^). Grey bars indicate missing data.

From the WGS data, MLSTs were determined for 943 (98 %) isolates. A total of 119 different sequence types (STs) were identified, the majority of the isolates (77 %) could be classified into one of 21 STs represented by ≥10 isolates (Fig. 1, Table S1), while 45 STs were represented by a single isolate. Clustering according to NG-MAST and the genome-based clustering methods, BAPS and PopPUNK, were found to be associated with the clustering of MLST (Fig. S2). NG-MAST and the genome-based clustering methods resulted in more clusters representing each ST (Table S2), and a few cases where multiple STs representing the same NG-MAST or genome-based cluster (Fig. S3). BAPS level 1 clustering was found with the highest association combined both as predictor and response variable to the MLST clusters (Fig. S2). A notable exception was the split of ST-1901 and ST-1893 into smaller clusters.

The phylogenetic relationship between all isolates is shown in Fig. 1, together with summarized patient data and phenotypic AST. Recombination events were identified covering an average of 129 558 nt (6 % of the genome) that were subsequently masked from the alignment (Fig. S5). In total, 11 310 SNP sites represented the variation in the data set after excluding possible recombination events.

STs represented by 10 or more isolates were stratified by sex (male vs. female), age group (<31 vs. ≥31 years), residency (greater Oslo vs. the rest of Norway) or phenotypic resistance (resistant vs. susceptible) using exact binomial test against the total average for each variable (Table S1). One ST (ST-8156) was significantly overrepresented in male patients, and three STs (ST-7359, ST-1901 and ST-1594) were significantly overrepresented in female patients compared to the total average (Table S1). Five STs consisted solely of isolates from male patients (Table S1). No STs were significantly associated with older than the median age of the patients (≥31 years old), but one ST (ST-7359) was found significantly associated with younger patients (Table S1). Two STs (ST-9363 and ST-1599) were significantly more likely encountered in patients from the greater Oslo region (Table S1). ST-1587 was significantly more likely isolated from urethra (Table S1). Stratification using genome-based clustering revealed similar patterns of associations with the corresponding ST clusters (Table S3).

Phenotypic resistance to the different antibiotics varied between the STs; ST-1587, ST-1588 and ST-1901 included many isolates resistant to several antibiotics, whereas ST-7359 and ST-8122 exhibited only a few isolates resistant to one or two antibiotics (Table S1). While six of the 21 most common STs included only CIP-sensitive isolates, eight STs included solely isolates that were resistant to CIP (Table S1). A similar, but less pronounced pattern was observed for PCN and TET, where the total number of resistant isolates was found to be close to a fourth of the total, and the individual STs exhibited either relatively high or relatively low number of resistant isolates (Table S1). CFM resistance was only observed in three STs (ST-1901, ST-7363 and ST-1893). AZM resistance was uncommon among the most abundant STs, but resistance was observed for isolates belonging to the ST-9363, ST-1901, ST-9143, ST-1580 and ST-1579 (Table S1). As described above, no phenotypic resistance was observed against CRO and SPX (Table 1 and S1).

### Multiple isolates from individual patients

**Fig. 2.**
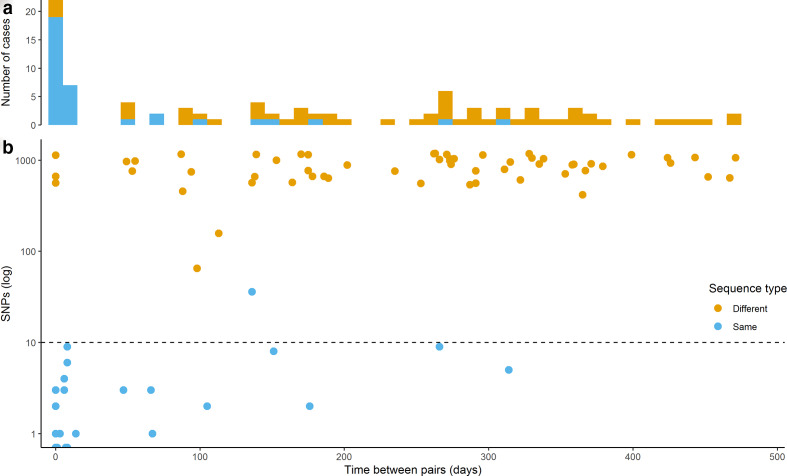
On the same *x*-axis showing time between pairs of isolates (in days): (a) the number of cases with either different or same ST, and (b) the pairwise SNP differences (log scale) between isolates.

From the 873 patients included in the study, 65 contributed two isolates and 10 contributed three isolates (Table 1). The majority of these isolates were sampled at different time points (122/160, 76 %), ranging from 1 to 471 days apart (Fig. 2, Table 3). The majority (110/122, 90 %) of the isolates from different time points were sampled more than 2 weeks apart, the average time between these collections was close to 7 months (208 days). The majority of the isolates sampled from the same patient on the same day were from different anatomical sites (28/38, 74 %), and belonged to the same ST (30/38, 79 %, Table 3). Three male patients were found to harbour clones belonging to different STs (ST-9363/ST-8156, ST-7827/ST-9362, ST-11516/ST-1588 with 561, 662, 1135 SNP differences, respectively) on the same sampling day at different anatomical sites. Indicating a possibility for either multiple infection in one sexual partner or infection acquired from more than one partner. Five pairs of isolates were sampled from the same anatomical site at the same time, all pairs belonged to the same ST, with an average of 1.4 SNP differences.

**Table 3.** Multiple isolates from individual patients

	No. of isolates	Different sample site	Same ST		Different ST	
			Fraction	SNPs (mean)	Fraction	SNPs (mean)
**Same day**	38	74 %	79 %	0.9	21 %	786
Different days*	122	26%	21 %	4.7	79 %	845

*1-471 days apart.

The majority of the isolates from patients sampled at different time points were from the same anatomical sampling site (90/122 74 %), and belonged to different STs (96/122, 79 %, Table 3). Three isolate pairs were found with an intermediate number of SNPs; one pair defined as the same ST (36 SNPs – ST-8156), whereas two pairs as different STs (65 and 157 SNPs, Fig. 2). Excluding isolates sampled less than 2 weeks apart, 39 patients (3.9 % of all patients), all males, were reinfected with a different clone within the study period and eight patients (0.8 % of all patients) were reinfected twice with the same clone. The proportion of reinfected patients with a new clone increased with time between sampling (i.e. 18/25, 72 % after 6 months and 45/54, 83 % after 1 year).

## Discussion

The observed demographics of the gonorrhea patients, young males from the urban region encompassing greater Oslo region (Table 1), correspond to typically reported risk groups [33]. Information about the sexual behaviour of the patients was not available and could thus not be linked to the isolates included in this study, however the majority of all cases notified with gonorrhea during the study period were MSM (55 and 65 % for 2016 and 2017, respectively, MSIS data). High male-to-female ratio as seen among the patients in Norway is strongly related to MSM behaviour [34], as well as the propensity to reside in urban settings [35, 36].

The distribution of STs among the Norwegian isolates was similar to that found in studies of isolates of European origin. Harris *et al.* [16] found 103 STs among 1054 isolates (approximately the same number as in the present study), with 23 STs including 10 or more isolates (compared to 21 in this study), and 35 STs represented by a single isolate (compared to 45 in this study). The observation of STs represented by a few or a single isolate in this study may indicate limited transmission within the Norwegian population, recent introductions, or unsampled patient populations. The most common STs observed in the present study match those found previously in Norway and Scandinavia (particularly, ST-1901), and in Western Europe and the USA (ST-9363 and ST-7363) [16, 37]. The most common STs observed in the present study, ST-8156 and ST-1599, appear to be relatively uncommon outside of Norway. A few observations of ST-8156 have been made in the UK (2014–2015) and Spain (2016), as well as some observations of ST-1599 in Kenya (2010–2015), as reported in the PubMLST database. ST-7359, while relatively common in Norway, has not been frequently observed in Europe. However, this ST is endemic in Japan and Australia [38, 39]. Two major groups of infected individuals can be drawn from the stratification of STs, one group likely consisting of MSM in their 30 s in the greater Oslo region (e.g. ST-8156, ST-9363, or ST-1599), and another group consisting of younger individuals including both males and females living outside the greater Oslo region (e.g. ST-7359, ST-1901, or ST-1588). Stratification of STs by AMR did not correspond to the transmission groups mentioned above. The concurrence of both highly resistant (e.g. ST-1901) and susceptible (e.g. ST-7359) STs circulating within the same demographic group indicates population dynamics of the bacteria (e.g. different selective advantages) allowing both phenotypes to coexist.

A few STs were represented by more than one genome-based cluster, the opposite; single genome-based clusters representing many STs were less common (Table S2, Fig. S3). The BAPS level 1 clusters were found to be closely associated with the ST clusters, with a few exceptions (e.g. splitting ST-1901 and ST-1893 into two clusters; Table S3).

Following international guidelines [40], the first-line therapy for gonorrhea in Norway consists of dual treatment using CRO and AZM. The phenotypic susceptibility of the isolates from Norway was similar to that observed elsewhere in Europe, North America and China [33, 41, 42]. No CRO resistance was detected in the isolates included in this study, and levels of CFM resistance was low (1%). Strong associations of antimicrobial resistance with specific clones was seen, especially for CIP and AZM. Overall, 100% CIP resistance was observed for ST-7827, as well as for the widespread ST-1901 and ST-7363 [16, 43], and less common STs, such as ST-8143, and ST-1587, Table S1). In the highly recombinant *N. gonorrhoeae *, point mutations may effectively be transferred between distantly related isolates through natural transformation or they may arise by independent *de novo* mutations. Both mechanisms will result in a polyphyletic distribution of the traits, i.e. CIP resistance acquired by single mutations of *gyrA* or *parC*. While England has experienced a long-term gonococcal outbreak with high-level (>256 mg l^−1^) AZM resistance among MSM caused by ST-9768 [44, 45], only four isolates with high-level AZM resistance were identified in our collection. All four isolates belonged to a different sequence type, ST-9363. The same 23S rRNA 2059A→G mutation observed in the outbreak strain in England [46] was found in three of our four isolates – these three were isolated from three patients sampled within 1 week. Widespread observations of PCN and TET resistance have been reported before [47]. While resistance to SPX is extremely rare [16, 48], and the drug could be an effective alternative in the treatment of uncomplicated cases, SPX is not very effective in eliminating the bacterium from the pharynx [49].

The genome-based AMR prediction of the 958 whole-genome-sequenced isolates revealed good match against the phenotypic resistance observations for four out of the seven antimicrobials tested, but predictions were poor for AZM, PCN and TET (Table 2). AZM and TET MIC values exhibited bimodal distributions, whereas PCN MIC values exhibited a unimodal distribution (Fig. S1). These distributions may represent complex interactions between mutations and phenotypes. With the exception of a few CIP-resistant isolates not predicted, and isolates not predicted in the ‘intermediate’ category against AZM and PCN (grey bars on the right panels in Fig. S1), the study does not indicate any undiscovered mutations (or genes) responsible for the observed phenotypic resistance (elevated MIC). The phenotypic SIR was based on the clinical breakpoints set by EUCAST, which is widely used and acknowledged – further development of the resistance databases is needed to improve the genotypic SIR predictions of AZM and PCN. Another solution may also be on the horizon, as several research groups are currently developing and training machine learning tools to correctly predict MIC values using genomic data.

Isolate-pairs with the same ST and low SNP differences (<10 SNPs) likely represent reinfections with the same clone after treatment. The time from infection to symptoms is relatively short (within a week) for gonorrhea [50, 51]. Including delay in seeking treatment (up to a week) [52, 53], one would normally expect no more than a few weeks from transmission to collection of isolate. All confirmed cases of gonorrhea in Norway are treated with CRO and AZM; as none of the isolates was resistant to CRO, inefficient treatment and persistent infection is therefore unlikely. The patient may be reinfected by an untreated contact, or the clone may be circulating with high prevalence in the population (or the patients’ sexual transmission network). The same results were observed using the presence of different ST or the SNP difference criteria (>60 SNP difference) described by De Silva *et al*. [54] to define reinfections of new clones. All isolate-pairs with same ST had <10 SNPs with the exception of one pair of isolates belonging to ST-8156 (Fig. 2) – this is the most abundant ST in the data set (Table S1). These two divergent ST-8156 isolates were likely representatives of two separate clusters circulating in Norway in the study period. The isolates that were sampled within a short time-frame (i.e. 2 weeks) may represent multiple sampling efforts prior to treatment, not reinfections (of the same clone). In the present dataset, one fourth of the patients with recurrent infections were found to be reinfected by the same clone, the likelihood of reinfection by the same clone (same ST) decreased over time (Fig. 2). Three patients were identified harbouring different clones simultaneously at different anatomical sites. All but one of these six isolates belonged to highly abundant STs; the exception was ST-9362 that was only found in eight patients in the data set. The proportion of patients with multiple infections caused by different clones was smaller in our study than in the one in Brighton, England, reported by De Silva *et al*. [54]. However, their study stretched over more than 4 years – twice that of the present study. Thus, our number of reinfected patients are likely underestimated, due to the limited study period.

### Conclusions

In conclusion, the cases of the pathogenic bacterium *N. gonorrhoeae* in Norway in 2016–2017 can be stratified roughly into two categories, one MSM group primarily in and around the capital, and one group represented by younger males and females from other localities in Norway. The distribution of the phenotypic AMR against some, but not all antimicrobials, follow that of the ST clusters. The predicted genotypic AMR using WGS data are shown to explain some of the observed SIR data. WGS data from isolates sampled from the same patients at different time points allow for distinction between reinfections of the same clone and new infections. The likelihood of observing reinfections of new clones increase with time between infections.

## Data Bibliography

Alfsnes *et al.* ENA. PRJEB32435 (2019).

## Supplementary Data

Supplementary material 1
