# Supplementary material for: Genomic epidemiology and population structure of Neisseria gonorrhoeae in Norway, 2016–2017
Source: Microb Genom. 2020 Mar 26;6(4):e000359. doi: 10.1099/mgen.0.000359 (PMC7276708; doi:10.1099/mgen.0.000359)
Supplement: Supplementary material 1 [file mgen-6-359-s001.pdf]

Table S1 – Patient data and phenotypic resistance for the most common sequence types (ST)

ST	Number of isolates*	Sex (probability male)	Sig. †	Age (median years)	Age (probability ≥31 years)	Sig. †	Residency (probability greater Oslo)	Sig. †	Infectious site (probability urethra)	Sig. †	CIP (probability resistant)	Sig.†	CEF (probability resistant)	CFM (probability resistant)	Sig. †	AZM (probability resistant)	Sig. †	PCN (probability resistant)	Sig. †	SPX (probability resistant)	TET (probability resistant)	Sig. †
8156	106	0.99	***	32	0.53		0.66		0.51		0.01	***	0	0		0.01	*	0.01	***	0	0.01	
9363	95	0.93		36	0.59		0.82	***	0.34		0.11	***	0	0		0.28	***	0	***	0	0.11	
7359	66	0.59	***	27	0.27	*	0.44		0.49		0	***	0	0		0.02		0	***	0	0.02	
1599	60	0.95		34	0.60		0.80	*	0.43		0	***	0	0		0		0	***	0	0.62	
1901	57	0.54	***	28	0.37		0.54		0.37		1	***	0	0.09	**	0.44	***	0.47	**	0	0.65	
7363	52	0.77		30	0.42		0.60		0.46		1	***	0	0.06		0.04		0.06	**	0	0.35	
8143	32	0.90		34	0.59		0.38		0.47		1	***	0	0		0.06		0.66	***	0	0.19	
7827	30	0.97		32	0.60		0.77		0.33		1	***	0	0		0		0.2		0	0.77	
1587	29	0.86		32	0.52		0.34		0.79	*	1	***	0	0		0		0.79	***	0	1	*
8122	28	0.89		27	0.29		0.82		0.54		0.04	***	0	0		0		0	*	0	0	
1588	26	0.65		25.5	0.31		0.42		0.50		1	***	0	0		0.04		0.73	***	0	0.92	
11428	19	1		31	0.47		0.74		0.37		0	***	0	0		0.21		0		0	0	
1584	17	1		34	0.59		0.88		0.35		0	***	0	0		0		0.12		0	0	
11516	17	1		36	0.53		0.65		0.59		0	***	0	0		0.06		0.47		0	0	
1596	13	0.85		32	0.54		0.77		0.62		0.08	**	0	0		0		0		0	0.07	
1893	13	0.92		26	0.15		0.69		0.23		0.31		0	0.08		0		0		0	0.31	
1580	12	0.58		25.5	0.17		0.58		0.67		0	*	0	0		0.83	***	0		0	0	
10314	12	1		36	0.67		0.75		0.58		1	***	0	0		0		0.08		0	0.17	
1579	11	1		29	0.36		0.45		0.45		0.36		0	0		0.55	**	0.45		0	0.64	
7822	11	0.82		31	0.45		0.45		0.36		1	***	0	0		0		0		0	0.55	
1594	9	0.33	*	23	0.11		0.33		0.33		0.11		0	0		0		0		0	0	
STs <10[i]	195	0.82		31	0.47		0.49		0.56		0.59	***	0	0		0.05		0.44	***	0	0.47	
NA	15	0.80		33	0.60		0.73	*	0.40		0.33		0	0		0.07		0.13		0	0.27	
Average		0.81		31			0.67		0.48		0.43		0	0.01		0.09		0.22		0	0.33	

**Multiple isolates belonging to the same ST isolated from the same patient are represented by a single entry, [i]STs represented by less than 10 isolates*

*†Sig. = exact binomial test of probability against the total average for each variable with multiple-comparison post-hoc correction using the Holm-Bonferroni method, *** = 0.001, ** = 0.01, * = 0.05*

CIP = ciprofloxacin, CEF = ceftriaxone, CFM = cefixime, AZM = azithromycin, PCN = penicillin g, SPX = spectinomycin, TET = tetracycline

Table S2 – Number of clusters and representative clusters using NGMAST and genome-based clustering methods; BAPS (level 1 & 2) and PopPUNK for the most common (N>10 isolates) sequence types (ST)

ST	Number of isolates	Number of NG-MAST clusters	Major NG-MAST (%)	Number of BAPS level 1 clusters	Major BAPS level 1 (%)	Number of BAPS level 2 clusters	Major BAPS level 2 (%)	Number of PopPUNK clusters	Major PopPUNK (%)
8156	114	10	5441 (51%)	3	19 (98%)	5	67 (79%)	23	1 (71%)
9363	99	14	17194 (67%)	1	7 (100%)	2	27 (98%)	17	2 (68%)
7359	67	6	4186 (85%)	1	13 (100%)	3	49 (61%)	9	3 (88%)
1599	61	5	11461 (48%)	1	6 (100%)	3	23 (80%)	14	5 (39%)
1901	58	21	NA (24%)	2	5 (84%)	3	16 (64%)	26	9 (24%)
7363	56	16	9184 (21%)	3	8 (73%)	5	31 (63%)	15	6 (39%)
8143	34	10	5624 (41%)	1	4 (100%)	4	11 (56%)	18	14 (26%)
7827	31	10	10386 (42%)	2	3 (94%)	3	9 (65%)	9	8 (52%)
1587	30	10	na (40%)	1	9 (100%)	2	33 (90%)	18	18 (27%)
8122	28	2	292 (96%)	1	2 (100%)	1	7 (100%)	2	4 (96%)
1588	26	8	na (65%)	1	16 (100%)	3	59 (46%)	14	13 (42%)
11428	19	6	298 (42%)	1	7 (100%)	2	27 (63%)	10	28 (32%)
1584	17	4	11744 (82%)	1	14 (100%)	3	52 (88%)	4	10 (82%)
11516	17	2	5793 (59%)	1	20 (100%)	3	71 (41%)	5	26 (35%)
1596	15	3	384 (80%)	2	22 (93%)	3	75 (80%)	4	12 (80%)
1893	14	4	8517 (43%)	2	15 (64%)	3	55 (43%)	5	23 (43%)
1580	12	5	18575 (50%)	1	7 (100%)	1	26 (100%)	6	30 (42%)
10314	12	4	16065 (42%)	1	5 (100%)	1	20 (100%)	3	19 (67%)
1579	11	7	21 (27%)	2	11 (73%)	3	41 (73%)	4	17 (73%)
7822	11	5	14994 (45%)	1	5 (100%)	2	15 (55%)	8	54 (18%)
1594	10	5	25 (30%)	1	21 (100%)	3	72 (50%)	6	49 (30%)
STs <10[i] N=45	201	74	NA	16	NA	48	NA	143	NA
NA	15	11	NA (25%)	9	19 (31%)	10	67 (31%)	14	NA (19 %)

[i]*STs represented by less than 10 isolates*

Table S3 – Patient data and phenotypic resistance for all BAPS level 1 clusters

BAPS level 1	Corresponding ST (proportion of cluster)	Number of isolates*	Sex (probability male)	Sig. †	Age (median years)	Age (probability ≥31 years)	Sig. †	Residency (probability greater Oslo)	Sig. †	Infectious site (probability urethra)	Sig. †	CIP (probability resistant)	Sig.†	CEF (probability resistant)	CFM (probability resistant)	Sig. †	AZM (probability resistant)	Sig. †	PCN (probability resistant)	Sig. †	SPX (probability resistant)	TET (probability resistant)	Sig. †
7	9363 (62%)	155	0.90		31	0.50		0.75	**	0.35	*	0.08	***	0	0		0.32	***	0	***	0	0.08	*
19	8156 (93%)	112	0.99	***	32	0.52		0.66		0.49		0.01	***	0	0		0.01	*	0	***	0	0	
5	1901 (45%)	109	0.73		30	0.45		0.61		0.44		0.98	***	0	0.07	***	0.25	***	0.28		0	0.50	
13	7359 (97%)	68	0.59	***	27	0.26	***	0.44		0.47		0.00	***	0	0		0.01		0	***	0	0.01	
6	1599 (100%)	60	0.95		34	0.6		0.80	*	0.43		0	***	0	0		0		0	***	0	0.62	
1	1925 (18%)	41	0.80		34	0.66		0.39		0.68		0.95	***	0	0		0	*	0.83	***	0	0.73	
8	7363 (93%)	41	0.80		34	0.56		0.63		0.41		0.98	***	0	0		0.05		0	**	0	0.32	
16	1588 (60%)	43	0.69		30	0.44		0.53		0.60		1	***	0	0		0.02		0.79	***	0	0.93	
4	8143 (85%)	38	0.92		33	0.55		0.34	*	0.50		1	***	0	0		0.05		0.61	***	0	0.16	
9	1587 (75%)	38	0.87		32	0.53		0.32	**	0.82	***	1	***	0	0		0		0.79	***	0	1.00	***
3	7827 (85%)	33	1	*	32	0.55		0.67		0.33		1	***	0	0		0		0.15		0	0.70	
10	1893 (16%)	30	0.75		27	0.33		0.47		0.67		0.47		0	0		0		0.33		0	0.73	
2	8122 (100%)	28	0.89		27	0.29		0.82		0.54		0.04	***	0	0		0		0	*	0	0	
14	1584 (65%)	26	0.96		36.5	0.65		0.77		0.38		0.12	***	0	0		0		0.19		0	0.12	
11	1579 (35%)	23	0.74		28	0.26		0.65		0.35		0.52		0	0		0.22		0.52	*	0	0.70	
20	11516 (74%)	23	0.96		31	0.48		0.65		0.52		0	***	0	0		0.04		0.48		0	0	
22	1596 (100%)	12	0.83		31.5	0.50		0.75		0.67		0	*	0	0		0		0		0	0	
17	8135 (50%)	11	0.50		29	0.36		0.27		0.45		0	*	0	0		0		0		0	0.09	
12	1901 (82%)	10	0.70		25.5	0.10		0.20		0.60		1	***	0	0		0.10		0.80	**	0	0.60	
21	1594 (100%)	9	0.33	*	23	0.11		0.33		0.33		0.11		0	0		0		0		0	0	
15	1893 (100%)	9	1		27	0.22		0.67		0.11		0		0	0.11		0		0		0	0	
18	11177 (100%)	6	0.80		27.5	0.33		0.67		0.83		0		0	0		0		0		0	0	

**Multiple isolates belonging to the same BAPS level 1 cluster isolated from the same patient are represented by a single entry*

*†Sig. = exact binomial test of probability against the total average for each variable with multiple-comparison post-hoc correction using the Holm-Bonferroni method, *** = 0.001, ** = 0.01, * = 0.05*

CIP = ciprofloxacin, CEF = ceftriaxone, CFM = cefixime, AZM = azithromycin, PCN = penicillin g, SPX = spectinomycin, TET = tetracycline

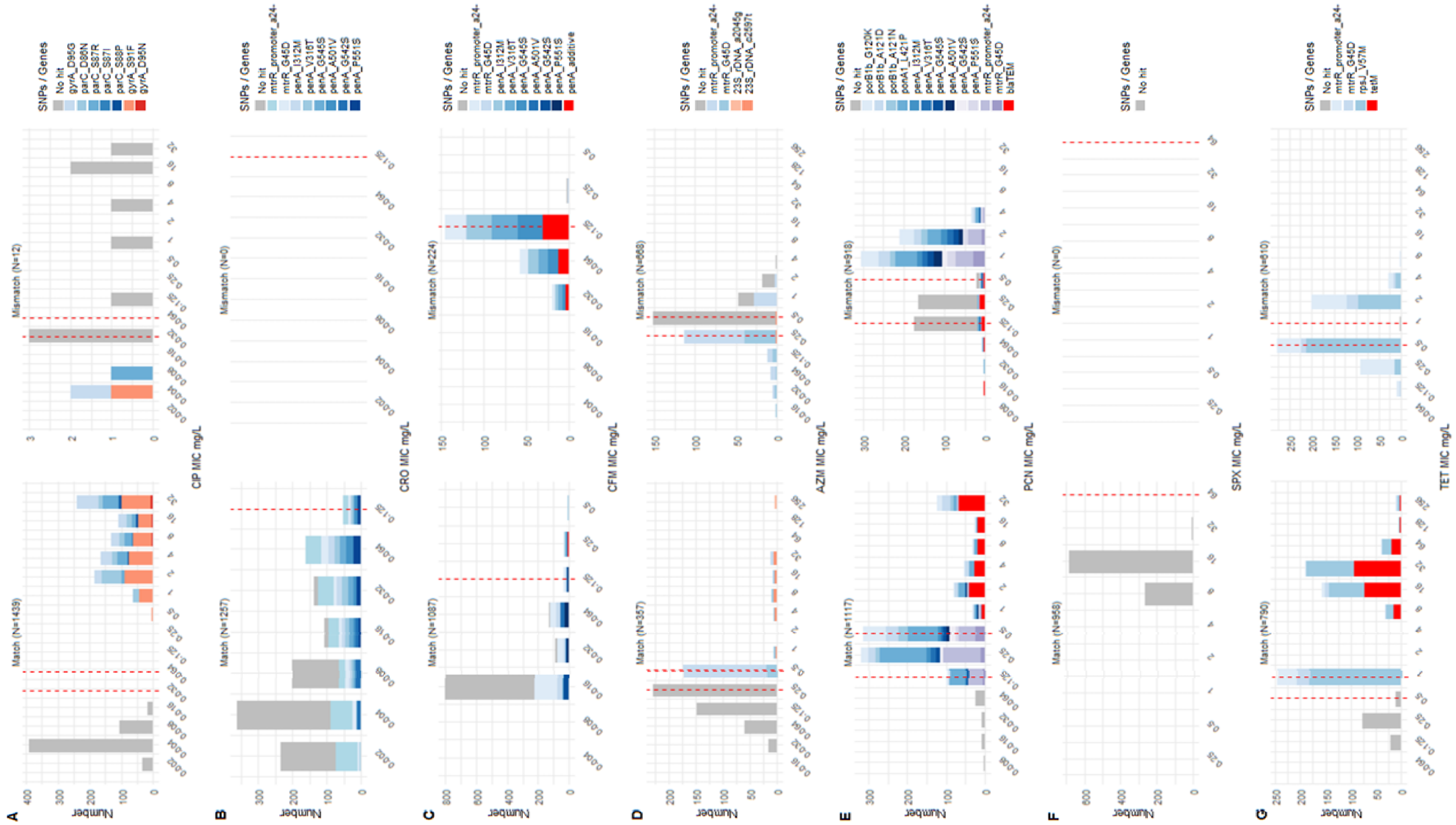

Fig. S1 – Bar charts showing the number of matches (left) and mismatches (right) between the phenotypic observations and genotypic predictions of antimicrobial resistance. Bars are colored according to hits in the Pathogenwatch AMR database, shades of blue represent hits scored as “intermediate”, shades of red “resistant”, and grey bars show isolates without any hits and consequently assumed as “susceptible”. Each panel (row) represent different antimicrobials, A – ciprofloxacin (CIP), B – ceftriaxone (CRO), C – cefixime (CFM), D – azithromycin (AZM), E – penicillin G (PCN), F – spectinomycin (SPX), G – tetracycline (TET). Red dashed lines represent the EUCAST breakpoint values for “susceptible”, “intermediate” and “resistant”. In the case of panel B (CRO), C (CFM) and F (SPX), EUCAST only separate between “susceptible” and “resistant”, predicted “intermediate” genotypes are designated as “susceptible” for these antimicrobials in the present study. Isolates may be represented by more than one mutation.

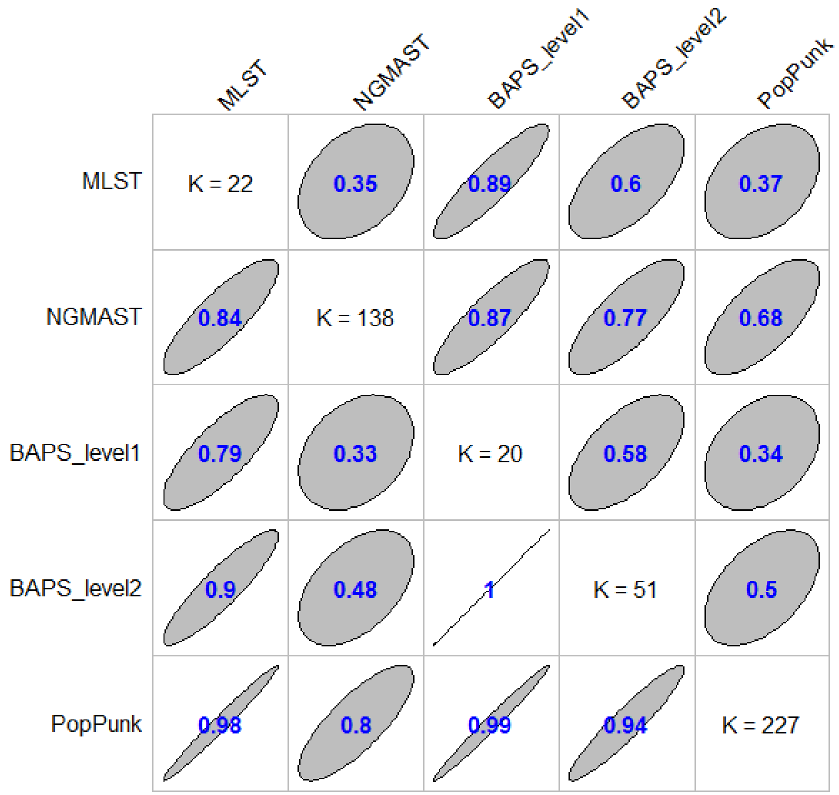

Fig. S2 – Goodman-Kruskal τ measuring the (asymmetric) association between categorical variables. The different clustering methods tested shown, predictors listed vertically and response variables listed horizontally, K represent the number of clusters for each method.

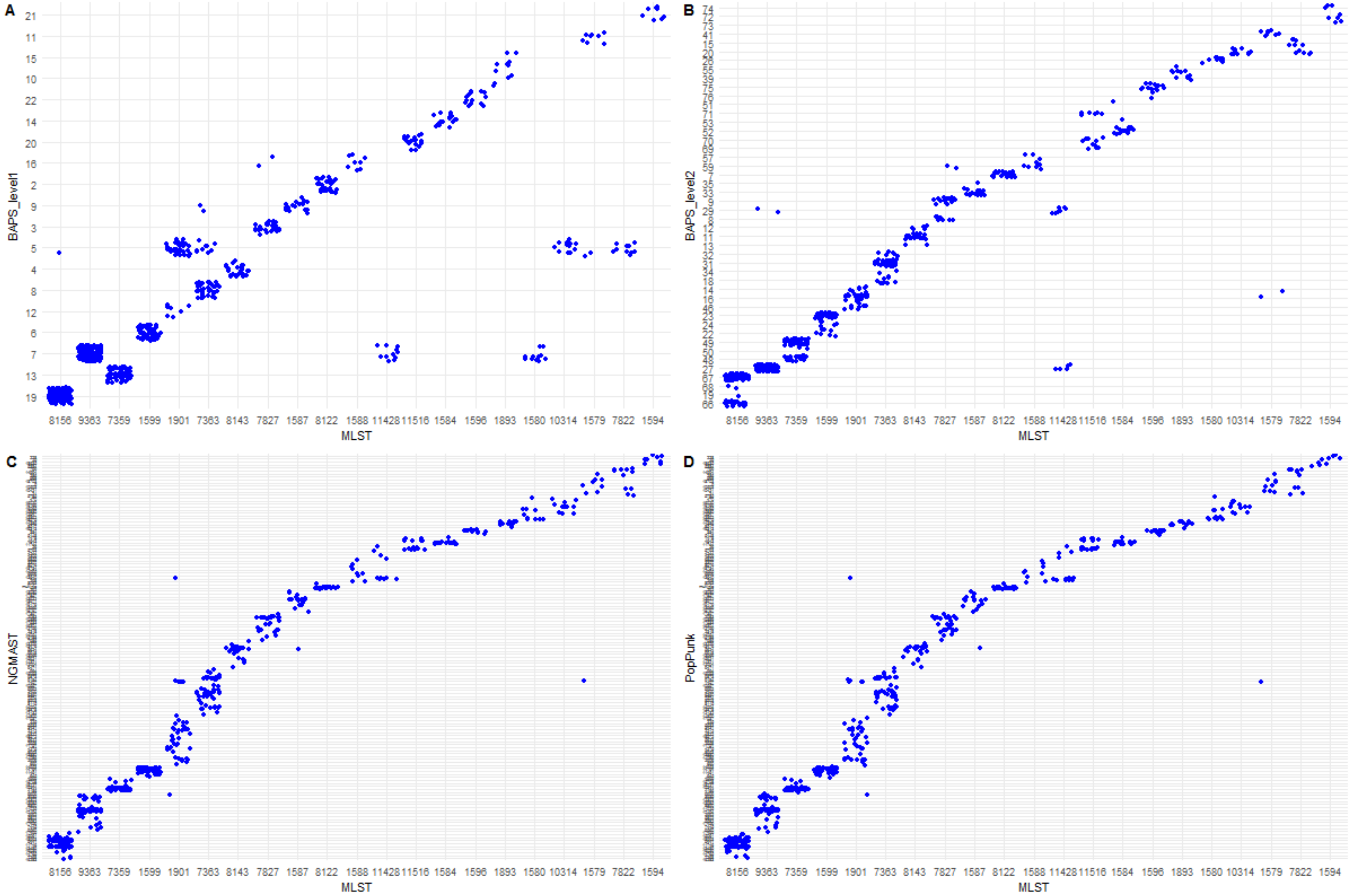

Fig S3 – Pairwise comparisons of the most common STs (represented by >10 isolates) and NGMAST (panel C), and whole-genome based clustering methods; BAPS level 1 (panel A) and level 2 (panel B), and PopPUNK (panel D).

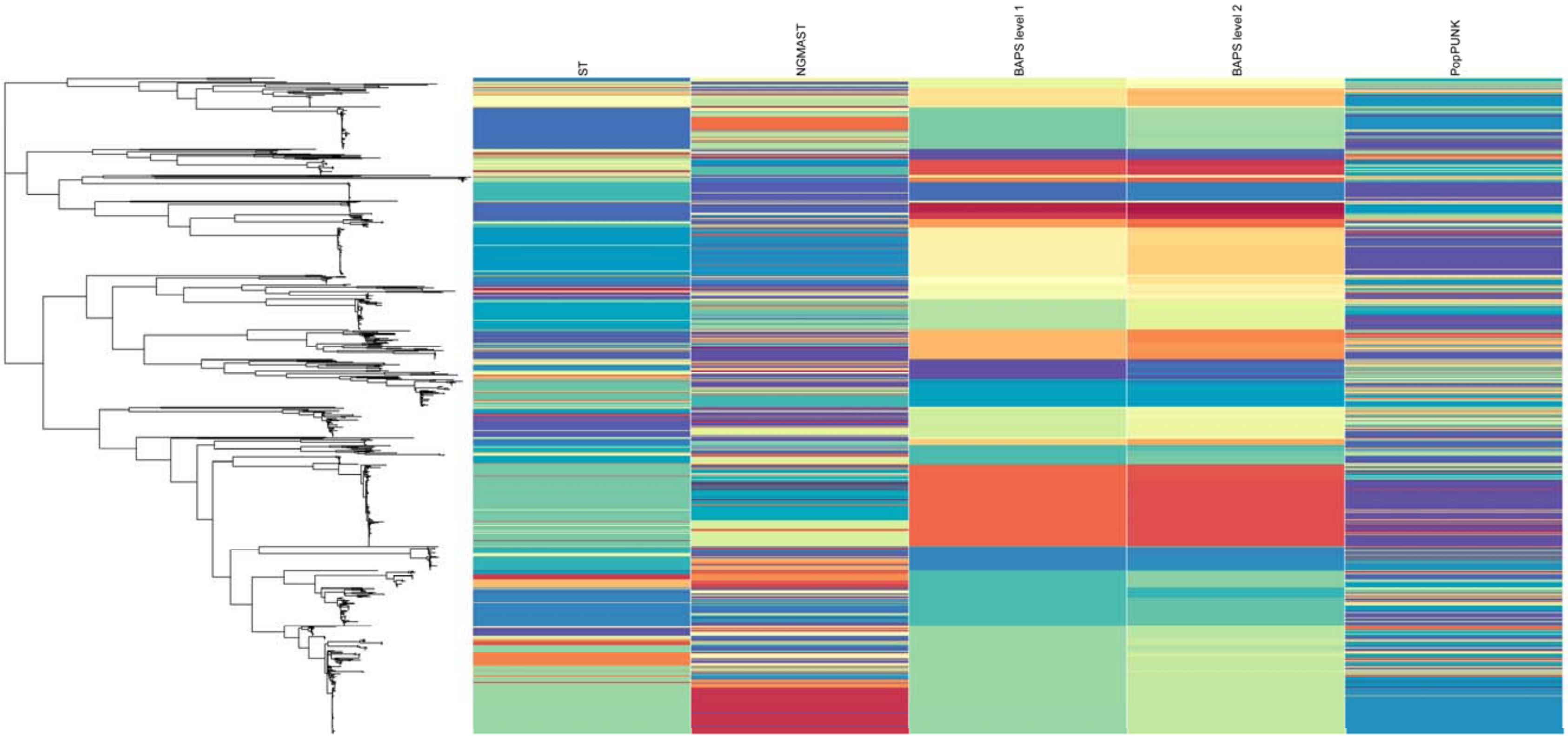

Fig. S4 – Maximum likelihood phylogeny of all available Norwegian *Neisseria gonorrhoeae* in the data set. Comparing sequence types (ST), NG-MAST, and two levels of BAPS clustering; level 1 & 2 (following the default settings in rhierBAPS; *max.depth=2, n.pop=number of isolates/5*), and PopPUNK.

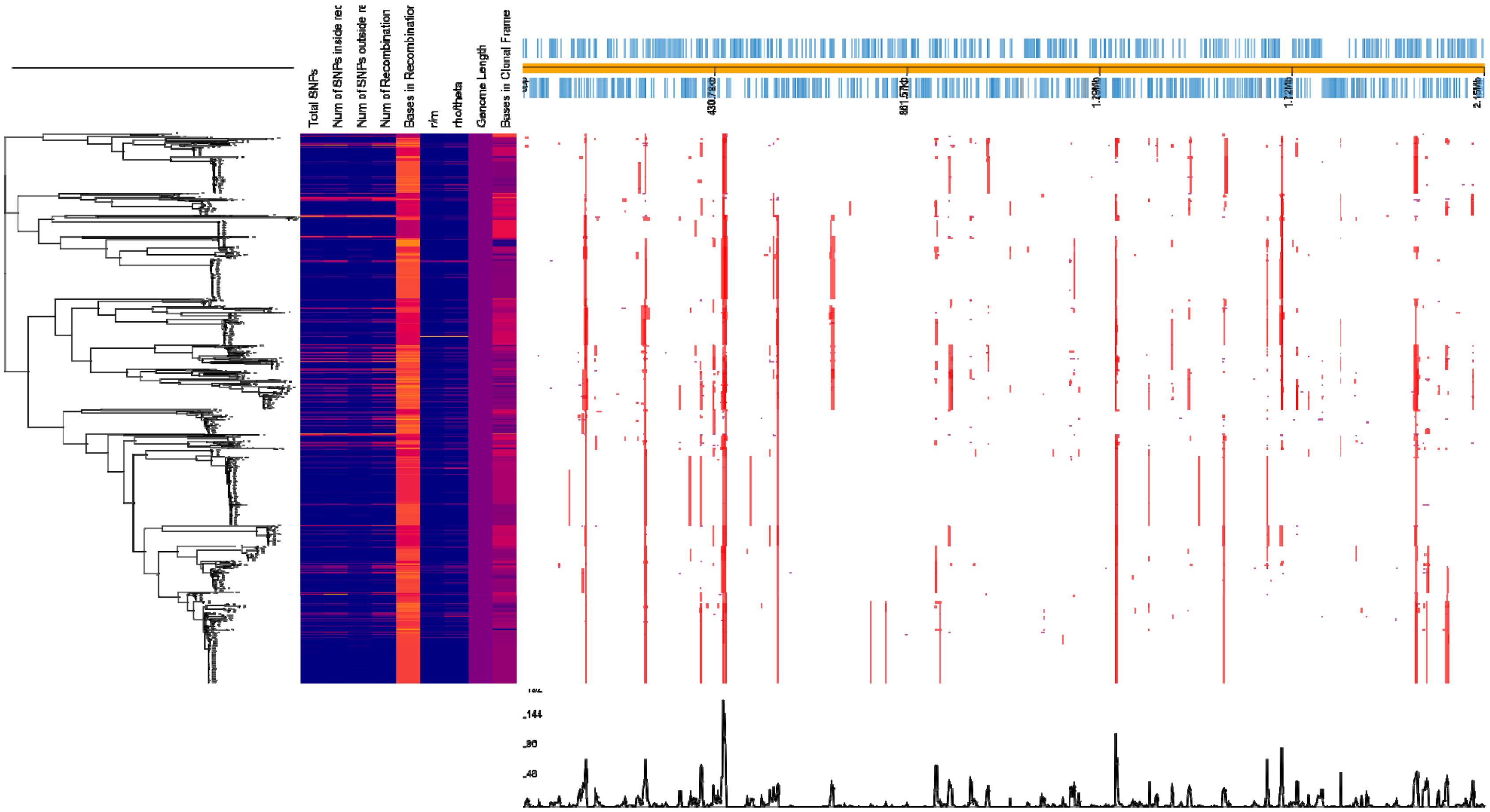

Fig. S5 – Maximum likelihood phylogeny of all available Norwegian *Neisseria gonorrhoeae* in the data set. Illustrating the detected and consequently removed recombination regions using Gubbins.

Supplementary table

Isolate names	Accession	CIP_MIC	CRO_MIC	CFM_MIC	AZM_MIC	PCN_MIC	SPX_MIC	TET_MIC
515386	SAMEA5608213	2	0.004	<0.016	0.125	32	16	0.5
515634	SAMEA5608214	0.004	0.008	<0.016	0.25	0.5	16	0.5
515640	SAMEA5608215	16	0.064	0.125	0.5	2	16	2
515707	SAMEA5608216	2	0.004	<0.016	0.25	0.25	16	0.5
515710	SAMEA5608217	4	0.016	0.032	0.5	1	8	1
516303	SAMEA5608218	0.004	0.004	<0.016	0.125	0.125	16	32
516308	SAMEA5608219	0.004	0.004	0.016	0.25	0.25	16	0.5
516428	SAMEA5608220	0.004	0.004	<0.016	0.25	0.25	8	0.5
516932	SAMEA5608221	0.004	0.008	0.016	0.25	0.25	8	0.5
517052	SAMEA5608222	>32	0.016	0.25	0.064	0.25	8	0.5
517960	SAMEA5608223	0.004	0.008	0.032	4	0.125	16	0.5
518063	SAMEA5608224	2	0.004	<0.016	0.5	32	16	8
518242	SAMEA5608225	4	0.008	0.032	0.5	0.125	16	16
518244	SAMEA5608226	0.004	0.004	<0.016	0.25	0.25	16	0.5
518613	SAMEA5608227	0.004	0.004	<0.016	4	0.125	16	0.5
518630	SAMEA5608228	0.004	0.004	<0.016	4	0.125	16	0.5
518644	SAMEA5608229	8	0.004	<0.016	0.032	4	16	0.5
518657	SAMEA5608230	8	0.008	0.032	0.064	0.125	16	0.5
518916	SAMEA5608231	>32	0.016	0.125	1	1	8	2
519014	SAMEA5608232	2	0.008	<0.016	0.125	32	16	32
519451	SAMEA5608233	0.004	0.008	0.016	0.25	0.25	16	0.5
519462	SAMEA5608234	0.004	0.004	<0.016	0.125	0.125	16	16
520479	SAMEA5608235	0.016	0.016	0.032	1	2	16	2
521112	SAMEA5608236	>32	0.032	0.064	0.5	4	16	2
521175	SAMEA5608237	4	0.016	0.064	0.25	0.25	8	16
523392	SAMEA5608238	0.004	0.002	<0.016	0.032	2	8	0.5
523399	SAMEA5608239	0.008	0.004	<0.016	0.5	0.25	16	0.25
523401	SAMEA5608240	4	0.004	<0.016	0.125	2	16	16
523471	SAMEA5608241	0.008	0.004	<0.016	0.25	0.25	16	0.25
523473	SAMEA5608242	0.004	0.016	0.032	0.25	0.5	8	1
523475	SAMEA5608243	2	0.004	<0.016	0.125	32	16	8
523848	SAMEA5608244	1	0.004	<0.016	0.25	0.25	16	8
523851	SAMEA5608245	0.004	0.004	<0.016	0.125	0.25	8	16
526504	SAMEA5608246	4	0.004	<0.016	0.25	0.25	16	16

529017	SAMEA5608247	>32	0.032	0.064	0.125	0.5	8	64
529019	SAMEA5608248	2	0.004	<0.016	0.064	32	16	0.5
529308	SAMEA5608249	0.004	0.064	0.25	0.25	0.5	8	0.5
529311	SAMEA5608250	4	0.064	0.064	0.25	1	16	1
529316	SAMEA5608251	8	0.016	0.125	0.032	0.125	8	0.25
529558	SAMEA5608252	32	0.032	0.125	0.5	1	8	2
529570	SAMEA5608253	0.008	0.004	<0.016	0.25	0.125	16	0.25
529771	SAMEA5608254	4	0.008	<0.016	0.25	>32	16	64
529775	SAMEA5608255	0.008	0.004	<0.016	0.25	0.125	16	0.25
529779	SAMEA5608256	0.004	0.004	<0.016	0.5	0.064	16	0.5
529782	SAMEA5608257	0.008	0.004	<0.016	0.25	0.125	16	0.25
529785	SAMEA5608258	1	0.008	<0.016	0.125	4	16	32
530056	SAMEA5608259	0.004	0.004	<0.016	0.125	0.125	8	0.5
530060	SAMEA5608260	4	0.002	<0.016	0.125	0.125	8	16
530591	SAMEA5608261	1	0.004	<0.016	0.064	4	16	32
530615	SAMEA5608262	0.002	0.002	<0.016	0.125	0.125	8	0.5
530801	SAMEA5608263	0.004	0.002	<0.016	0.5	0.064	16	0.5
531027	SAMEA5608264	>32	0.064	0.25	0.5	2	16	2
531223	SAMEA5608265	0.004	0.004	<0.016	0.5	0.125	16	0.5
531362	SAMEA5608266	0.008	0.004	<0.016	0.25	0.25	16	0.25
531664	SAMEA5608267	2	0.008	0.016	0.064	>32	16	16
531684	SAMEA5608268	1	0.004	<0.016	0.125	16	16	16
532249	SAMEA5608269	0.004	0.004	<0.016	0.125	1	8	0.5
532439	SAMEA5608270	0.004	<0.002	<0.016	0.064	0.016	16	0.125
532644	SAMEA5608271	0.004	0.008	0.016	0.125	0.25	16	0.5
533224	SAMEA5608272	0.004	<0.002	<0.016	0.25	0.016	16	0.25
533451	SAMEA5608273	1	0.008	<0.016	0.125	1	16	32
534206	SAMEA5608274	0.004	0.004	<0.016	0.125	2	16	16
534211	SAMEA5608275	0.008	0.004	<0.016	0.25	0.25	16	0.5
534219	SAMEA5608276	4	0.002	<0.016	0.032	0.125	8	16
534235	SAMEA5608277	8	<0.002	<0.016	0.064	0.125	8	32
534273	SAMEA5608278	>32	0.032	0.032	0.5	4	16	2
534278	SAMEA5608279	>32	0.125	0.032	0.5	4	16	2
534283	SAMEA5608280	0.008	0.008	<0.016	0.5	0.5	16	0.5
534289	SAMEA5608281	8	0.016	0.032	0.5	1	16	1
534293	SAMEA5608282	>32	0.032	0.032	0.5	2	16	2

534296	SAMEA5608283	>32	0.032	0.032	0.5	2	16	2
534321	SAMEA5608284	0.016	0.032	0.016	1	2	16	2
534323	SAMEA5608285	2	0.008	0.016	0.125	16	16	32
534448	SAMEA5608286	0.004	<0.002	<0.016	0.125	0.032	16	0.5
534452	SAMEA5608287	>32	0.008	<0.016	0.25	4	16	32
535164	SAMEA5608288	2	0.016	<0.016	0.125	>32	16	32
535479	SAMEA5608289	>32	0.032	0.032	1	2	16	4
535699	SAMEA5608290	2	0.004	<0.016	0.125	16	8	0.5
536036	SAMEA5608291	16	0.064	0.125	1	2	16	4
536169	SAMEA5608292	0.004	0.002	<0.016	0.125	2	16	16
536289	SAMEA5608293	0.5	0.008	<0.016	0.5	>32	16	32
536292	SAMEA5608294	0.004	<0.002	<0.016	0.25	0.032	16	0.5
536447	SAMEA5608295	0.004	<0.002	0.016	0.25	0.064	16	0.5
536453	SAMEA5608296	0.008	0.008	<0.016	0.25	0.25	16	0.5
536598	SAMEA5608297	0.008	0.008	<0.016	0.25	0.25	8	0.5
536688	SAMEA5608298	>32	0.064	0.125	1	2	16	2
536928	SAMEA5608299	1	0.008	<0.016	0.25	>32	16	32
537058	SAMEA5608300	4	0.008	0.016	0.064	8	16	32
537159	SAMEA5608301	4	0.016	<0.016	0.5	1	16	4
537170	SAMEA5608302	>32	0.032	0.25	0.064	0.5	8	0.5
537175	SAMEA5608303	4	0.016	0.016	1	1	16	1
537844	SAMEA5608304	16	0.032	0.125	0.064	0.5	8	0.5
538145	SAMEA5608305	>32	0.064	0.064	0.5	4	16	2
538150	SAMEA5608306	2	0.004	<0.016	0.125	0.25	16	32
538405	SAMEA5608307	>32	0.016	0.016	0.5	4	16	64
538583	SAMEA5608308	1	0.008	<0.016	0.064	4	16	32
538826	SAMEA5608309	2	0.004	<0.016	0.125	>32	16	32
539414	SAMEA5608310	0.008	0.004	<0.016	0.5	0.5	16	0.5
539437	SAMEA5608311	1	0.008	<0.016	0.032	4	16	32
539457	SAMEA5608312	1	0.008	<0.016	0.064	4	16	32
539460	SAMEA5608313	>32	0.004	<0.016	0.125	4	16	32
539526	SAMEA5608314	>32	0.125	0.032	1	2	16	8
539885	SAMEA5608315	>32	0.064	0.125	1	2	16	2
540315	SAMEA5608316	0.008	0.004	<0.016	0.5	2	16	1
540484	SAMEA5608317	>32	0.032	0.064	1	1	16	4
540998	SAMEA5608318	0.008	0.008	<0.016	0.5	0.25	16	0.5

541000	SAMEA5608319	1	0.008	<0.016	0.064	4	16	32
541002	SAMEA5608320	16	0.008	<0.016	0.5	>32	16	64
541004	SAMEA5608321	1	0.008	<0.016	0.064	8	16	32
541213	SAMEA5608322	0.008	0.008	<0.016	0.25	0.25	16	0.25
541220	SAMEA5608323	0.008	0.008	<0.016	0.25	0.25	8	0.25
541996	SAMEA5608324	0.004	0.008	<0.016	0.064	0.125	16	0.125
542012	SAMEA5608325	2	0.008	<0.016	0.5	>32	16	0.5
542364	SAMEA5608326	2	0.008	0.016	0.125	8	16	32
542478	SAMEA5608327	0.008	0.004	<0.016	0.125	0.125	0.25	8
542677	SAMEA5608328	2	0.008	<0.016	0.5	0.5	16	1
542781	SAMEA5608329	>32	0.064	0.125	2	2	16	4
542783	SAMEA5608330	0.004	0.008	0.016	8	0.5	16	1
542849	SAMEA5608331	1	0.004	<0.016	0.125	2	8	32
542910	SAMEA5608332	>32	0.032	0.125	0.064	0.5	16	1
543452	SAMEA5608333	0.008	0.008	<0.016	0.25	0.25	16	0.25
543557	SAMEA5608334	0.008	0.004	<0.016	0.25	0.125	8	0.25
543561	SAMEA5608335	0.004	0.004	<0.016	8	1	16	1
543568	SAMEA5608336	4	0.004	<0.016	0.25	0.25	8	1
544352	SAMEA5608337	0.004	0.004	<0.016	0.5	0.125	16	0.5
544354	SAMEA5608338	0.008	0.008	<0.016	0.5	0.25	16	0.5
544360	SAMEA5608339	0.004	0.008	<0.016	0.25	1	16	0.25
544362	SAMEA5608340	0.004	0.002	<0.016	0.25	1	16	0.5
544365	SAMEA5608341	0.004	0.004	<0.016	0.125	2	8	0.5
544467	SAMEA5608342	8	0.016	<0.016	0.25	0.5	16	1
544558	SAMEA5608343	2	0.004	<0.016	0.064	16	16	16
546852	SAMEA5608344	0.008	0.002	<0.016	0.5	0.25	16	0.5
547917	SAMEA5608345	0.008	0.008	<0.016	1	0.25	16	1
547920	SAMEA5608346	>32	0.032	0.125	1	2	8	2
549070	SAMEA5608347	1	0.004	<0.016	0.064	8	16	32
549082	SAMEA5608348	2	0.008	<0.016	0.064	>32	16	32
549092	SAMEA5608349	>32	0.032	0.125	1	2	16	2
549528	SAMEA5608350	0.008	0.008	<0.016	0.25	0.25	16	1
549760	SAMEA5608351	>32	0.032	0.125	1	2	16	2
549872	SAMEA5608352	>32	0.032	0.064	0.5	0.5	16	2
550339	SAMEA5608353	8	0.008	<0.064	0.5	>32	16	64
551037	SAMEA5608354	>32	0.032	0.125	1	2	16	2

551147	SAMEA5608355	0.002	<0.002	<0.016	0.064	0.008	16	8
551154	SAMEA5608356	>32	0.032	0.064	0.5	1	16	2
551585	SAMEA5608357	0.004	0.002	<0.016	0.5	0.5	16	0.25
551600	SAMEA5608358	0.008	0.004	<0.016	0.5	0.5	16	0.25
551975	SAMEA5608359	0.004	0.004	<0.016	0.25	0.125	8	0.5
551979	SAMEA5608360	2	0.032	0.064	0.25	0.5	8	1
552145	SAMEA5608361	4	<0.002	<0.016	0.032	0.125	16	8
552460	SAMEA5608362	0.004	0.004	<0.016	0.5	0.25	16	0.5
552468	SAMEA5608363	0.008	0.008	0.016	0.25	0.5	16	0.5
552472	SAMEA5608364	1	0.004	<0.016	0.25	2	16	16
552474	SAMEA5608365	0.004	0.004	<0.016	0.25	0.25	16	0.5
552476	SAMEA5608366	>32	0.064	0.064	0.5	1	16	2
552521	SAMEA5608367	0.004	<0.002	<0.016	0.125	0.032	16	0.125
552524	SAMEA5608368	0.004	0.008	0.016	0.25	0.5	16	0.5
552526	SAMEA5608369	0.004	0.008	0.016	0.25	0.5	16	0.5
552553	SAMEA5608370	0.004	0.008	0.016	0.25	0.5	16	0.5
552560	SAMEA5608371	8	0.125	0.125	0.25	2	8	1
552577	SAMEA5608372	>32	0.125	0.25	1	4	16	2
552580	SAMEA5608373	8	0.004	<0.016	0.064	4	16	8
552586	SAMEA5608374	1	0.008	<0.016	0.25	2	16	16
552588	SAMEA5608375	0.016	0.016	0.016	0.5	1	16	1
552591	SAMEA5608376	0.004	0.004	<0.016	1	0.25	16	0.5
552594	SAMEA5608377	0.004	0.004	0.016	0.5	0.5	16	0.25
552602	SAMEA5608378	0.004	<0.002	<0.016	0.5	0.032	16	0.25
552606	SAMEA5608379	0.004	0.008	0.016	0.125	0.25	8	0.5
552610	SAMEA5608380	8	0.004	<0.016	0.25	2	8	16
552659	SAMEA5608381	0.004	0.008	0.016	0.25	0.25	16	1
552703	SAMEA5608382	8	0.008	0.016	0.25	>32	16	32
552828	SAMEA5608383	0.004	0.008	<0.016	0.25	0.25	16	0.5
552832	SAMEA5608384	>32	0.032	0.032	0.5	>32	8	128
552835	SAMEA5608385	0.016	0.016	<0.016	0.5	1	8	1
552837	SAMEA5608386	4	0.004	<0.016	0.25	0.25	8	16
552840	SAMEA5608387	0.008	0.008	<0.016	0.25	0.25	16	0.25
552852	SAMEA5608388	0.004	0.008	<0.016	0.125	0.25	16	0.5
552854	SAMEA5608389	>32	0.032	0.032	0.25	0.5	8	2
552856	SAMEA5608390	>32	0.016	<0.016	0.25	>32	8	>256

552858	SAMEA5608391	4	0.032	0.032	0.5	0.5	8	1
552860	SAMEA5608392	>32	0.032	0.032	0.25	0.5	8	2
552866	SAMEA5608393	4	0.008	<0.016	0.25	0.5	16	16
552868	SAMEA5608394	0.008	0.008	<0.016	0.5	0.25	16	0.25
552872	SAMEA5608395	0.004	0.008	<0.016	0.25	0.5	16	16
552874	SAMEA5608396	>32	0.125	0.5	0.25	4	16	2
552878	SAMEA5608397	>32	0.064	0.125	0.25	1	8	2
552885	SAMEA5608398	4	0.004	<0.016	0.25	2	8	8
552888	SAMEA5608399	0.004	0.002	<0.016	0.25	4	8	0.25
552891	SAMEA5608400	0.004	0.008	<0.016	0.125	0.25	8	0.5
552894	SAMEA5608401	0.004	0.004	<0.016	0.032	0.25	8	0.5
552896	SAMEA5608402	8	0.008	<0.016	0.125	2	8	8
552910	SAMEA5608403	0.004	0.008	<0.016	0.25	0.25	32	1
553545	SAMEA5608404	1	0.016	0.008	0.016	1	16	2
553566	SAMEA5608405	>32	0.016	0.125	0.032	0.25	8	0.25
553571	SAMEA5608406	>32	0.032	0.064	32	1	16	2
553579	SAMEA5608407	>32	0.064	0.125	0.5	2	16	1
553585	SAMEA5608408	0.008	0.004	<0.016	0.25	0.25	16	0.25
553597	SAMEA5608409	0.004	0.004	<0.016	0.25	0.125	16	0.5
553599	SAMEA5608410	>32	0.032	0.064	0.25	1	16	>256
553601	SAMEA5608411	0.004	0.004	<0.016	0.125	0.25	8	16
553603	SAMEA5608412	16	0.016	0.016	0.25	>32	16	1
553615	SAMEA5608413	0.004	0.008	0.016	0.5	0.25	16	0.5
553617	SAMEA5608414	0.004	0.004	<0.016	0.25	0.25	16	0.5
553619	SAMEA5608415	4	0.004	<0.016	0.032	1	8	16
553621	SAMEA5608416	2	0.004	<0.016	0.125	>32	8	16
553623	SAMEA5608417	0.004	0.004	<0.016	0.25	0.25	16	1
553630	SAMEA5608418	0.004	0.004	<0.016	0.5	2	16	0.5
553633	SAMEA5608419	2	0.004	<0.016	0.125	16	16	16
553637	SAMEA5608420	2	0.004	<0.016	0.032	2	8	8
553647	SAMEA5608421	0.004	0.004	<0.016	0.25	0.5	8	0.5
553651	SAMEA5608422	0.004	0.008	0.016	8	0.5	16	0.5
553653	SAMEA5608423	0.008	0.008	<0.016	0.5	0.25	8	0.5
553711	SAMEA5608424	16	0.004	<0.016	0.064	2	8	16
553714	SAMEA5608425	0.004	0.008	<0.016	0.25	0.25	16	0.5
553718	SAMEA5608426	0.004	0.004	<0.016	0.25	0.125	8	1

553721	SAMEA5608427	0.016	0.016	0.016	2	2	16	4
553724	SAMEA5608428	>32	0.064	0.032	0.25	1	8	>256
553727	SAMEA5608429	16	0.008	0.032	0.25	16	8	64
553730	SAMEA5608430	>32	0.016	0.125	0.064	0.25	8	0.5
553734	SAMEA5608431	2	0.008	<0.016	0.5	>32	16	0.5
553739	SAMEA5608432	0.004	0.004	<0.016	0.064	0.25	8	0.5
553742	SAMEA5608433	0.004	0.004	<0.016	0.5	0.25	16	0.25
553744	SAMEA5608434	0.004	0.016	0.032	0.25	0.25	16	0.5
553751	SAMEA5608435	0.004	0.008	<0.016	0.5	0.25	16	0.5
553753	SAMEA5608436	0.016	0.064	0.032	1	2	16	2
553756	SAMEA5608437	4	0.002	<0.016	0.5	>32	32	1
553760	SAMEA5608438	>32	0.032	0.125	32	1	16	2
553821	SAMEA5608439	4	0.008	<0.016	0.25	>32	16	16
553823	SAMEA5608440	>32	0.125	0.25	0.25	2	16	4
553826	SAMEA5608441	1	0.008	0.016	0.25	2	16	32
553829	SAMEA5608442	0.008	0.008	<0.016	0.25	0.25	16	0.25
553832	SAMEA5608443	0.004	0.016	0.016	0.25	0.25	16	1
553887	SAMEA5608444	0.004	<0.002	<0.016	0.25	0.125	8	0.25
554395	SAMEA5608445	>32	0.064	0.125	32	1	16	4
554470	SAMEA5608446	2	0.004	0.016	0.064	2	16	32
554554	SAMEA5608447	0.008	0.004	<0.016	0.125	0.125	8	0.125
554556	SAMEA5608448	4	0.008	<0.016	0.064	16	16	16
554561	SAMEA5608449	0.008	0.004	<0.016	0.25	2	16	0.5
554563	SAMEA5608450	2	0.008	<0.016	0.125	32	16	32
554565	SAMEA5608451	2	0.008	<0.016	0.25	0.25	16	16
554567	SAMEA5608452	2	0.004	<0.016	0.25	>32	16	0.5
554569	SAMEA5608453	0.004	0.016	0.064	0.25	0.125	8	0.25
554584	SAMEA5608454	0.004	0.008	<0.016	0.25	0.25	16	0.5
554586	SAMEA5608455	1	0.004	<0.016	0.064	16	8	0.5
554590	SAMEA5608456	4	0.002	<0.016	0.25	0.125	16	0.5
554601	SAMEA5608457	0.004	0.004	<0.016	0.25	0.25	8	0.25
554613	SAMEA5608458	0.5	0.008	<0.016	0.125	4	16	32
554621	SAMEA5608459	0.004	0.002	<0.016	0.032	4	16	16
554624	SAMEA5608460	0.004	0.004	0.016	0.5	0.25	8	0.5
554631	SAMEA5608461	8	0.064	0.064	0.5	2	16	2
554635	SAMEA5608462	0.004	0.004	<0.016	0.125	0.125	8	32

554641	SAMEA5608463	0.004	0.008	0.016	0.5	0.125	8	0.5
554646	SAMEA5608464	0.004	0.008	0.016	0.064	0.125	8	0.125
554649	SAMEA5608465	8	0.064	0.064	0.5	1	8	2
554653	SAMEA5608466	4	0.064	0.064	0.25	0.5	8	2
554674	SAMEA5608467	4	0.016	<0.016	0.25	0.5	8	2
554677	SAMEA5608468	4	0.008	<0.016	0.5	0.25	16	16
554680	SAMEA5608469	0.004	0.016	0.032	16	1	16	1
554684	SAMEA5608470	0.004	0.008	<0.016	0.25	1	8	1
554689	SAMEA5608471	0.004	0.008	0.016	16	1	16	1
554729	SAMEA5608472	>32	0.064	0.125	0.5	1	8	2
554735	SAMEA5608473	0.004	0.002	<0.016	0.5	2	8	1
554737	SAMEA5608474	8	0.032	0.032	0.5	0.5	8	1
554739	SAMEA5608475	16	0.016	0.125	0.064	0.25	8	0.5
554741	SAMEA5608476	0.004	0.004	<0.016	0.125	0.125	8	0.5
554791	SAMEA5608477	0.004	0.008	<0.016	0.25	0.25	16	0.5
554793	SAMEA5608478	2	0.004	<0.016	0.25	0.25	16	0.5
554795	SAMEA5608479	0.008	0.008	<0.016	0.125	0.25	8	0.25
554797	SAMEA5608480	0.004	0.008	<0.016	0.125	0.25	8	0.5
554799	SAMEA5608481	0.004	0.004	<0.016	0.5	0.25	8	1
554801	SAMEA5608482	0.004	0.008	<0.016	0.125	0.25	8	0.5
554804	SAMEA5608483	0.004	0.008	<0.016	0.25	0.25	16	0.5
554806	SAMEA5608484	0.004	0.016	0.032	0.5	0.5	16	0.5
554813	SAMEA5608485	0.004	<0.002	<0.016	0.125	0.016	8	0.064
554816	SAMEA5608486	0.004	0.008	<0.016	0.25	0.5	16	16
555119	SAMEA5608487	>32	0.064	0.125	1	2	16	2
555122	SAMEA5608488	0.004	0.004	<0.016	0.25	0.25	8	32
555491	SAMEA5608489	0.008	0.016	0.064	0.25	0.25	16	0.25
555495	SAMEA5608490	>32	0.032	0.032	1	0.5	16	2
555497	SAMEA5608491	0.032	0.016	<0.016	2	0.5	16	2
555500	SAMEA5608492	0.004	<0.002	<0.016	0.25	0.125	16	1
555504	SAMEA5608493	0.004	0.004	<0.016	0.125	0.125	8	0.25
555507	SAMEA5608494	16	0.032	0.064	0.25	0.25	2	1
555509	SAMEA5608495	16	0.032	0.25	0.032	0.25	8	0.25
555523	SAMEA5608496	0.004	0.008	<0.016	8	1	16	1
555548	SAMEA5608497	>32	0.125	0.25	1	2	16	2
555559	SAMEA5608498	0.008	0.008	<0.016	0.25	0.25	16	0.5

555561	SAMEA5608499	>32	0.064	0.125	16	2	16	1
555564	SAMEA5608500	16	0.032	0.032	0.25	0.5	16	1
555572	SAMEA5608501	0.008	0.004	<0.016	0.25	0.25	16	1
555585	SAMEA5608502	>32	0.032	0.064	0.25	0.5	16	1
555589	SAMEA5608503	2	0.004	<0.016	0.25	>32	16	1
555607	SAMEA5608504	0.008	0.008	<0.016	0.25	0.25	16	0.5
555609	SAMEA5608505	8	0.032	0.032	0.5	0.5	16	2
555614	SAMEA5608506	0.004	<0.002	<0.016	0.5	0.25	16	0.25
555617	SAMEA5608507	0.008	0.004	<0.016	0.25	0.25	16	1
555812	SAMEA5608508	>32	0.016	<0.016	>256	0.25	16	2
555814	SAMEA5608509	0.004	0.008	<0.016	2	0.125	16	1
555816	SAMEA5608510	>32	0.064	0.064	0.5	0.5	16	4
555818	SAMEA5608511	0.004	0.004	<0.016	0.5	0.25	16	0.5
555820	SAMEA5608512	0.004	0.008	<0.016	0.25	0.25	8	1
555823	SAMEA5608513	0.008	0.008	<0.016	0.25	0.25	16	0.25
555827	SAMEA5608514	4	0.004	<0.016	0.064	2	16	32
555830	SAMEA5608515	4	0.008	<0.016	0.25	>32	16	1
555832	SAMEA5608516	8	0.004	<0.016	0.5	0.125	16	0.5
555834	SAMEA5608517	8	0.016	0.016	0.25	0.5	16	1
555841	SAMEA5608518	0.004	0.004	<0.016	1	0.125	8	0.5
555845	SAMEA5608519	0.004	0.008	<0.016	0.5	0.125	16	1
555855	SAMEA5608520	0.004	0.008	<0.016	0.25	0.25	16	1
555861	SAMEA5608521	0.004	<0.002	<0.016	0.125	0.032	8	0.125
555869	SAMEA5608522	0.004	0.004	<0.016	0.125	0.064	8	0.125
555876	SAMEA5608523	0.008	0.004	<0.016	0.25	0.125	8	32
555897	SAMEA5608524	0.008	0.008	<0.016	0.25	0.25	16	1
555899	SAMEA5608525	>32	0.032	0.032	0.25	0.5	16	2
555919	SAMEA5608526	0.008	0.004	<0.016	1	0.25	16	1
555924	SAMEA5608527	16	0.032	0.032	0.25	0.5	8	2
556567	SAMEA5608528	0.004	0.004	<0.016	0.25	0.25	16	0.5
556826	SAMEA5608529	4	0.032	0.032	0.25	0.25	8	1
556830	SAMEA5608530	0.004	0.004	<0.016	0.5	0.125	16	0.5
556832	SAMEA5608531	2	0.008	<0.016	0.5	0.25	16	0.5
556835	SAMEA5608532	0.004	0.008	<0.016	0.5	0.125	16	0.5
556837	SAMEA5608533	0.004	<0.002	<0.016	0.25	0.016	8	0.064
556840	SAMEA5608534	0.008	0.008	<0.016	0.125	0.25	8	0.25

556843	SAMEA5608535	2	0.008	0.032	0.125	16	16	32
556846	SAMEA5608536	0.004	<0.002	<0.016	0.5	0.25	16	0.125
556850	SAMEA5608537	0.004	0.008	<0.016	0.25	0.25	16	0.5
556853	SAMEA5608538	0.004	0.004	<0.016	0.25	0.064	16	0.5
556857	SAMEA5608539	0.004	0.004	<0.016	0.5	0.125	16	0.5
556860	SAMEA5608540	0.004	0.004	<0.016	0.5	0.25	16	0.25
556862	SAMEA5608541	0.004	0.002	<0.016	0.125	0.125	16	16
556874	SAMEA5608542	8	0.064	0.064	0.5	1	16	2
556876	SAMEA5608543	0.008	0.004	<0.016	0.125	0.125	16	0.25
556880	SAMEA5608544	0.004	0.004	<0.016	0.5	0.125	16	0.5
556882	SAMEA5608545	8	0.032	0.032	0.25	0.5	16	1
556884	SAMEA5608546	16	0.064	0.064	0.25	0.5	16	1
556888	SAMEA5608547	8	0.032	0.032	0.25	0.5	16	1
556891	SAMEA5608548	4	0.016	<0.016	0.25	1	8	0.5
556896	SAMEA5608549	1	0.004	<0.016	0.032	>32	16	0.5
556902	SAMEA5608550	0.004	0.002	<0.016	0.25	0.125	16	0.5
556909	SAMEA5608551	0.004	0.004	<0.016	0.064	2	8	0.25
557175	SAMEA5608552	0.016	0.032	0.032	1	2	16	4
557177	SAMEA5608553	16	0.064	0.125	0.5	0.5	16	2
557179	SAMEA5608554	0.004	0.004	<0.016	0.25	2	8	1
557753	SAMEA5608555	0.008	0.008	<0.016	0.25	0.25	32	1
558222	SAMEA5608556	2	0.004	<0.016	0.064	>32	16	16
558650	SAMEA5608557	0.004	0.002	<0.016	0.25	0.125	16	0.5
558652	SAMEA5608558	2	0.008	<0.016	0.125	32	16	32
558902	SAMEA5608559	0.008	0.004	<0.016	0.5	0.125	16	0.25
558906	SAMEA5608560	>32	0.032	0.032	0.5	0.5	16	2
559226	SAMEA5608561	2	0.008	<0.016	0.064	16	16	16
559699	SAMEA5608562	1	0.008	0.016	0.064	16	16	32
559716	SAMEA5608563	0.008	0.004	<0.016	0.5	0.125	16	1
559721	SAMEA5608564	0.008	0.008	<0.016	0.5	0.25	16	0.25
560534	SAMEA5608565	0.008	0.004	<0.016	0.25	0.125	16	0.25
560546	SAMEA5608566	0.004	0.004	<0.016	0.5	0.125	16	0.5
560558	SAMEA5608567	0.004	0.008	<0.016	0.25	0.25	16	0.25
560631	SAMEA5608568	0.008	0.004	<0.016	0.125	0.125	16	0.25
560639	SAMEA5608569	0.004	0.002	<0.016	0.25	2	16	0.5
560642	SAMEA5608570	0.004	0.004	<0.016	0.5	0.125	16	1

560645	SAMEA5608571	4	0.016	<0.016	0.5	1	16	1
560870	SAMEA5608572	0.008	0.004	<0.016	0.25	0.25	16	64
560974	SAMEA5608573	0.004	<0.002	<0.016	0.25	0.032	16	0.25
560978	SAMEA5608574	0.004	0.004	<0.016	0.5	0.125	16	1
560980	SAMEA5608575	4	0.008	<0.016	0.125	16	16	16
560982	SAMEA5608576	0.008	0.004	<0.016	0.25	0.125	16	1
561156	SAMEA5608577	16	0.064	0.125	0.25	1	8	2
561285	SAMEA5608578	0.004	0.004	<0.016	0.064	>32	16	1
561287	SAMEA5608579	0.008	0.004	<0.016	0.5	0.25	16	0.5
561473	SAMEA5608580	4	0.008	<0.016	0.25	>32	8	0.5
561732	SAMEA5608581	0.004	0.004	<0.016	0.5	0.125	16	1
561738	SAMEA5608582	2	0.004	<0.016	0.064	8	16	32
561752	SAMEA5608583	0.004	0.004	<0.016	0.5	0.125	16	0.5
561755	SAMEA5608584	0.004	0.004	<0.016	0.5	0.125	16	1
561760	SAMEA5608585	0.008	<0.002	<0.016	0.5	0.25	16	0.25
561767	SAMEA5608586	>32	0.064	0.125	0.5	2	16	1
561772	SAMEA5608587	>32	0.032	0.064	0.5	0.5	16	1
561776	SAMEA5608588	0.008	0.004	<0.016	0.5	0.25	16	0.25
561995	SAMEA5608589	>32	0.032	0.032	0.5	0.5	16	1
562099	SAMEA5608590	0.004	0.002	<0.016	0.5	0.125	16	0.25
562554	SAMEA5608591	2	<0.002	<0.016	0.032	16	16	32
562564	SAMEA5608592	1	0.004	<0.016	0.064	4	16	32
562568	SAMEA5608593	>32	0.064	0.064	0.25	0.5	8	1
562571	SAMEA5608594	8	0.008	<0.016	0.25	>32	16	32
562687	SAMEA5608595	4	0.004	<0.016	0.125	0.25	16	32
562691	SAMEA5608596	0.004	0.004	<0.016	0.25	0.25	16	1
562694	SAMEA5608597	0.004	0.004	<0.016	0.5	0.125	8	0.5
562697	SAMEA5608598	1	0.016	0.032	0.25	2	16	16
562700	SAMEA5608599	0.004	0.004	<0.016	0.25	0.25	16	32
562702	SAMEA5608600	0.004	0.004	<0.016	0.5	0.25	16	0.25
562927	SAMEA5608601	>32	0.032	0.032	1	0.5	16	2
562969	SAMEA5608602	0.004	0.004	<0.016	0.5	0.25	16	0.5
562974	SAMEA5608603	4	0.032	0.064	0.125	4	16	32
563148	SAMEA5608604	0.004	0.004	<0.016	0.5	0.25	16	0.25
563150	SAMEA5608605	8	0.032	0.032	0.5	0.5	16	1
563153	SAMEA5608606	0.004	0.004	<0.016	0.5	0.25	16	0.25

563155	SAMEA5608607	0.004	0.004	<0.016	0.5	0.125	16	1
563160	SAMEA5608608	0.004	0.004	<0.016	0.5	0.125	16	1
563192	SAMEA5608609	0.004	0.004	<0.016	0.125	0.25	16	32
563194	SAMEA5608610	0.004	0.004	<0.016	0.5	0.125	16	0.5
563196	SAMEA5608611	0.004	0.004	<0.016	0.5	0.25	16	0.25
563198	SAMEA5608612	0.004	0.004	<0.016	0.125	0.125	16	0.5
563203	SAMEA5608613	0.004	0.004	<0.016	0.5	0.25	16	0.125
563210	SAMEA5608614	0.004	0.004	<0.016	0.5	0.125	16	0.5
563253	SAMEA5608615	16	0.004	<0.016	0.125	2	8	16
563896	SAMEA5608616	0.004	0.032	0.064	0.25	1	16	1
563898	SAMEA5608617	2	0.004	<0.016	0.5	0.25	16	0.5
563908	SAMEA5608618	>32	0.016	0.016	0.5	1	16	2
563915	SAMEA5608619	16	0.032	0.25	0.25	0.5	8	0.5
564102	SAMEA5608620	0.004	0.004	<0.016	0.125	0.125	16	32
564104	SAMEA5608621	0.004	0.004	<0.016	0.5	0.125	16	1
564106	SAMEA5608622	0.008	0.004	<0.016	0.25	0.25	16	0.25
564538	SAMEA5608623	0.004	0.004	<0.016	1	0.125	16	0.5
564544	SAMEA5608624	0.004	0.004	<0.016	0.5	0.25	16	0.25
564548	SAMEA5608625	0.004	0.008	<0.016	0.5	0.25	16	0.5
564704	SAMEA5608626	0.004	0.004	<0.016	1	0.25	16	1
564715	SAMEA5608627	0.008	0.004	<0.016	0.25	0.25	16	0.125
564717	SAMEA5608628	4	0.004	<0.016	0.125	0.25	16	16
564719	SAMEA5608629	2	0.004	<0.016	0.125	0.25	16	32
564721	SAMEA5608630	0.004	<0.002	<0.016	0.5	0.125	8	0.25
564723	SAMEA5608631	>32	0.032	0.032	0.5	0.25	16	1
564725	SAMEA5608632	0.008	0.004	<0.016	0.125	0.25	16	0.25
564734	SAMEA5608633	< 0.002	0.004	<0.016	0.5	0.25	16	1
564736	SAMEA5608634	2	0.004	<0.016	0.25	>32	16	0.5
564776	SAMEA5608635	0.008	0.004	<0.016	0.25	0.25	16	0.25
564779	SAMEA5608636	4	0.004	<0.016	0.125	>32	16	32
565100	SAMEA5608637	0.004	0.004	<0.016	0.5	0.25	16	0.125
565102	SAMEA5608638	0.004	0.004	<0.016	0.5	0.125	16	0.5
565144	SAMEA5608639	8	0.064	0.064	0.5	1	16	2
565162	SAMEA5608640	0.004	0.004	<0.016	0.5	0.125	16	0.5
565166	SAMEA5608641	0.004	0.004	<0.016	1	0.125	16	0.5
565168	SAMEA5608642	0.004	0.004	<0.016	1	0.125	16	0.5

565172	SAMEA5608643	0.004	0.004	<0.016	1	0.25	16	0.5
565338	SAMEA5608644	1	0.008	<0.016	0.125	0.25	16	16
565457	SAMEA5608645	0.004	0.004	<0.016	0.5	0.25	16	0.25
565460	SAMEA5608646	0.008	0.004	<0.016	0.25	0.25	16	0.5
565462	SAMEA5608647	>32	0.064	0.064	0.5	0.5	16	2
565464	SAMEA5608648	8	0.008	<0.016	0.25	>32	16	64
565707	SAMEA5608649	0.004	0.002	<0.016	0.25	0.125	8	0.5
565709	SAMEA5608650	0.004	0.004	<0.016	0.25	0.125	16	0.5
565753	SAMEA5608651	0.008	0.008	<0.016	0.25	0.25	16	0.25
565757	SAMEA5608652	0.008	0.008	<0.016	0.25	0.25	8	0.25
565760	SAMEA5608653	2	0.004	<0.016	0.5	0.25	16	0.5
565763	SAMEA5608654	0.008	0.004	<0.016	0.125	0.125	8	0.125
565874	SAMEA5608655	16	0.064	0.064	0.5	0.5	16	2
566172	SAMEA5608656	8	0.008	<0.016	0.25	0.25	16	64
566481	SAMEA5608657	0.004	0.004	<0.016	0.25	0.125	16	1
566483	SAMEA5608658	0.004	0.004	<0.016	0.25	0.25	16	0.5
566485	SAMEA5608659	0.004	0.004	<0.016	0.5	0.25	16	0.5
566760	SAMEA5608660	8	0.064	0.125	0.5	1	16	2
566763	SAMEA5608661	>32	0.064	0.064	0.5	0.5	16	1
566769	SAMEA5608662	0.004	0.004	<0.016	0.5	0.25	16	0.5
566806	SAMEA5608663	0.008	0.008	<0.016	0.25	0.25	16	0.25
566809	SAMEA5608664	0.008	0.008	<0.016	0.25	0.25	16	0.25
567052	SAMEA5608665	0.008	0.008	<0.016	0.25	0.25	16	0.25
567058	SAMEA5608666	1	0.008	<0.016	0.125	0.25	16	32
567063	SAMEA5608667	0.004	0.008	<0.016	0.25	0.125	16	0.5
567065	SAMEA5608668	0.008	0.004	<0.016	0.25	0.125	16	0.25
567117	SAMEA5608669	1	0.016	0.016	0.25	8	16	64
567119	SAMEA5608670	0.008	0.008	<0.016	0.25	0.125	16	0.25
567121	SAMEA5608671	0.004	<0.002	<0.016	0.5	0.032	16	0.25
567125	SAMEA5608672	4	0.004	<0.016	0.5	>32	32	1
567315	SAMEA5608673	0.008	0.004	<0.016	0.25	0.125	16	0.25
567322	SAMEA5608674	2	0.004	<0.016	0.125	4	8	32
567326	SAMEA5608675	0.004	0.002	<0.016	0.5	0.125	16	0.5
567332	SAMEA5608676	16	0.016	0.064	0.5	0.5	16	2
567334	SAMEA5608677	0.016	0.008	<0.016	0.25	0.25	16	0.25
567341	SAMEA5608678	0.004	0.004	<0.016	0.5	0.125	16	0.25

567354	SAMEA5608679	0.016	0.008	<0.016	0.25	0.25	16	0.25
567369	SAMEA5608680	0.004	0.004	<0.016	0.125	0.125	8	32
567377	SAMEA5608681	0.004	0.004	<0.016	0.5	0.064	16	1
567380	SAMEA5608682	0.004	0.004	<0.016	0.5	0.25	16	0.5
567383	SAMEA5608683	0.004	0.004	<0.016	0.25	0.25	16	32
567385	SAMEA5608684	0.004	0.008	<0.016	0.5	0.25	16	0.5
567616	SAMEA5608685	0.004	0.004	<0.016	1	0.25	16	1
567623	SAMEA5608686	0.004	0.004	<0.016	0.25	0.25	16	1
567752	SAMEA5608687	16	0.032	0.032	0.25	0.5	16	1
567930	SAMEA5608688	2	0.004	<0.016	0.064	4	16	32
567933	SAMEA5608689	4	0.016	0.016	0.5	>32	16	64
568533	SAMEA5608690	16	0.125	0.125	0.25	1	8	1
568535	SAMEA5608691	0.008	0.004	<0.016	0.125	0.125	8	0.25
568538	SAMEA5608692	8	0.032	0.032	0.25	0.5	16	1
568626	SAMEA5608693	0.004	0.004	<0.016	0.25	0.25	16	1
568630	SAMEA5608694	0.004	0.002	<0.016	0.5	0.125	8	0.5
568633	SAMEA5608695	0.004	0.004	<0.016	1	0.125	16	0.5
568642	SAMEA5608696	0.004	0.004	<0.016	0.5	0.125	16	0.5
568645	SAMEA5608697	0.004	0.004	<0.016	0.5	0.125	16	0.5
568824	SAMEA5608698	0.008	0.008	<0.016	0.25	0.25	16	0.25
568826	SAMEA5608699	8	0.016	<0.016	0.5	1	16	1
568828	SAMEA5608700	0.004	0.004	<0.016	0.5	0.125	16	1
568905	SAMEA5608701	0.004	0.004	<0.016	0.5	0.25	8	0.25
569223	SAMEA5608702	>32	0.032	0.032	0.125	8	16	64
569225	SAMEA5608703	0.008	0.004	<0.016	0.5	0.25	16	0.25
569227	SAMEA5608704	0.004	0.004	<0.016	0.5	0.125	16	1
569261	SAMEA5608705	0.004	0.002	<0.016	1	0.25	16	1
569368	SAMEA5608706	0.004	0.004	<0.016	0.25	0.25	16	1
569370	SAMEA5608707	0.004	0.008	<0.016	0.5	0.25	16	0.5
569374	SAMEA5608708	0.004	0.004	<0.016	0.125	0.125	16	0.125
569376	SAMEA5608709	0.004	0.004	<0.016	0.5	0.25	16	0.25
569456	SAMEA5608710	0.004	0.004	<0.016	0.5	0.125	8	1
569792	SAMEA5608711	0.008	<0.002	<0.016	0.25	2	8	0.5
570002	SAMEA5608712	0.008	0.004	<0.016	0.125	0.125	8	0.125
570005	SAMEA5608713	0.008	0.004	<0.016	0.125	0.125	16	0.25
570007	SAMEA5608714	8	0.032	0.064	0.5	1	16	2

570009	SAMEA5608715	0.004	<0.002	<0.016	0.5	0.125	16	1
570384	SAMEA5608716	1	0.004	<0.016	0.064	4	8	32
570835	SAMEA5608717	0.008	0.004	<0.016	0.25	0.25	16	0.25
570963	SAMEA5608718	2	0.004	<0.016	0.25	>32	16	32
571444	SAMEA5608719	0.008	0.004	<0.016	0.25	0.25	16	0.5
571505	SAMEA5608720	>32	0.032	0.064	32	0.5	16	2
571509	SAMEA5608721	0.004	0.004	<0.016	0.25	0.5	8	0.125
571516	SAMEA5608722	0.004	0.002	<0.016	1	0.125	16	0.5
571518	SAMEA5608723	0.004	<0.002	<0.016	0.5	0.064	8	0.5
571521	SAMEA5608724	0.004	0.004	<0.016	0.25	0.25	16	0.5
571533	SAMEA5608725	0.008	0.004	<0.016	0.125	0.25	8	16
571535	SAMEA5608726	0.004	0.004	<0.016	0.5	0.25	8	0.5
571541	SAMEA5608727	0.004	0.004	<0.016	0.5	0.125	16	0.5
571543	SAMEA5608728	0.004	0.002	<0.016	0.25	0.125	16	0.25
571545	SAMEA5608729	4	0.032	0.032	0.5	0.5	16	2
571547	SAMEA5608730	0.004	<0.002	<0.016	0.125	0.125	8	32
571549	SAMEA5608731	0.004	<0.002	<0.016	0.25	0.125	8	32
571551	SAMEA5608732	0.004	0.004	<0.016	0.5	0.25	16	1
571553	SAMEA5608733	0.008	0.008	<0.016	0.5	0.25	16	0.25
571555	SAMEA5608734	0.008	0.004	<0.016	0.5	0.5	16	0.5
571557	SAMEA5608735	0.008	0.004	<0.016	0.25	0.25	16	1
571559	SAMEA5608736	0.008	0.004	<0.016	0.5	0.25	16	1
571795	SAMEA5608737	0.008	0.004	<0.016	0.5	0.25	16	0.25
571800	SAMEA5608738	0.008	0.004	<0.016	0.25	0.25	16	0.25
571802	SAMEA5608739	0.004	0.004	<0.016	0.5	0.125	16	0.5
572276	SAMEA5608740	0.004	0.004	<0.016	0.5	0.125	16	0.5
572278	SAMEA5608741	4	0.008	0.016	0.125	2	8	16
572280	SAMEA5608742	8	0.008	<0.016	0.25	>32	16	0.5
572710	SAMEA5608743	0.004	0.004	<0.016	0.125	2	16	32
572803	SAMEA5608744	1	0.004	<0.016	0.064	>32	8	16
572805	SAMEA5608745	0.008	<0.002	<0.016	0.25	0.25	16	64
572813	SAMEA5608746	0.004	<0.002	<0.016	0.125	0.125	16	32
572919	SAMEA5608747	1	0.002	<0.016	0.125	16	8	32
573089	SAMEA5608748	0.004	0.002	<0.016	0.25	0.25	16	0.5
573092	SAMEA5608749	0.004	0.004	<0.016	0.5	0.25	16	0.25
573136	SAMEA5608750	0.004	0.004	<0.016	1	0.25	16	1

573138	SAMEA5608751	0.004	0.004	<0.016	0.25	0.125	8	0.25
573321	SAMEA5608752	0.004	0.002	<0.016	0.5	0.125	16	0.5
573324	SAMEA5608753	0.008	0.004	<0.016	0.25	0.25	8	0.25
573338	SAMEA5608754	32	0.064	0.125	0.5	1	16	2
573398	SAMEA5608755	0.004	<0.002	0.016	0.5	0.25	16	0.25
573406	SAMEA5608756	0.004	0.004	<0.016	0.5	0.25	16	0.5
573414	SAMEA5608757	0.004	0.004	<0.016	0.5	0.25	16	1
573422	SAMEA5608758	0.5	0.002	<0.016	0.064	16	8	8
573425	SAMEA5608759	16	0.032	0.125	1	1	16	2
573427	SAMEA5608760	8	0.008	<0.016	0.5	0.5	16	1
573495	SAMEA5608761	0.004	0.002	<0.016	0.125	0.25	8	0.25
573567	SAMEA5608762	0.004	0.002	<0.016	0.5	0.125	16	0.5
573713	SAMEA5608763	2	0.002	<0.016	0.032	8	8	16
573718	SAMEA5608764	2	0.002	<0.016	0.125	0.125	16	8
573721	SAMEA5608765	2	0.008	0.016	0.125	>32	16	16
573969	SAMEA5608766	2	0.004	<0.016	0.064	>32	8	32
573972	SAMEA5608767	>32	0.032	0.032	32	2	8	2
573974	SAMEA5608768	0.004	0.004	<0.016	0.5	0.25	16	0.5
573976	SAMEA5608769	0.004	0.004	<0.016	0.5	0.25	16	0.5
573978	SAMEA5608770	>32	0.032	0.064	0.5	0.5	16	2
573980	SAMEA5608771	0.004	0.002	<0.016	0.5	0.25	16	0.25
573982	SAMEA5608772	0.002	0.004	<0.016	0.125	0.125	8	32
574133	SAMEA5608773	0.008	0.004	<0.016	0.25	0.25	16	1
574506	SAMEA5608774	8	0.008	<0.016	0.25	0.5	16	2
574623	SAMEA5608775	0.004	0.002	<0.016	0.25	0.25	16	1
574772	SAMEA5608776	1	0.004	0.016	0.125	>32	16	16
574837	SAMEA5608777	0.004	0.004	<0.016	0.25	0.25	16	32
574847	SAMEA5608778	0.004	0.002	<0.016	0.5	0.125	16	1
574850	SAMEA5608779	0.004	0.002	<0.016	0.5	0.125	16	0.5
574853	SAMEA5608780	1	0.016	0.032	0.25	2	16	16
574859	SAMEA5608781	0.004	0.004	0.016	0.5	0.25	16	1
574861	SAMEA5608782	4	0.004	0.016	0.032	4	8	16
575409	SAMEA5608783	>32	0.032	0.125	2	1	16	2
575440	SAMEA5608784	0.004	0.002	<0.016	0.064	0.016	8	0.25
575772	SAMEA5608785	0.004	<0.002	<0.016	0.5	0.064	16	1
575775	SAMEA5608786	0.004	0.002	<0.016	0.5	0.125	16	0.5

575779	SAMEA5608787	>32	0.016	0.032	0.25	8	16	64
575784	SAMEA5608788	0.004	0.002	<0.016	0.5	0.125	16	1
575786	SAMEA5608789	1	0.016	0.032	0.25	1	16	32
575788	SAMEA5608790	0.004	0.004	<0.016	0.5	0.25	16	1
575792	SAMEA5608791	8	0.008	0.016	0.5	1	16	1
575794	SAMEA5608792	0.004	0.008	<0.016	0.25	0.5	16	0.5
575796	SAMEA5608793	0.008	0.004	<0.016	0.5	0.125	16	0.5
575878	SAMEA5608794	1	0.002	<0.016	0.064	8	16	16
575939	SAMEA5608795	0.004	0.004	<0.016	0.5	0.25	16	0.25
576043	SAMEA5608796	8	0.004	<0.016	8	>32	16	64
576045	SAMEA5608797	4	0.004	<0.016	0.25	0.25	16	0.5
576176	SAMEA5608798	0.008	0.004	<0.016	0.25	0.25	16	0.25
576450	SAMEA5608799	0.125	<0.016	0.004	0.125	0.125	16	0.5
576564	SAMEA5608800	0.016	0.008	<0.016	4	0.5	16	2
576566	SAMEA5608801	0.004	0.004	<0.016	0.125	0.125	16	32
576568	SAMEA5608802	0.004	0.004	<0.016	0.25	0.25	8	0.5
577003	SAMEA5608803	0.002	<0.002	<0.016	0.25	0.064	8	0.25
577007	SAMEA5608804	4	0.008	<0.016	0.5	0.5	16	0.5
577012	SAMEA5608805	8	0.008	<0.016	0.25	0.5	16	0.5
577023	SAMEA5608806	0.004	0.004	<0.016	0.25	0.25	16	0.5
577121	SAMEA5608807	0.004	0.002	<0.016	0.125	0.064	8	0.125
577124	SAMEA5608808	4	0.016	<0.016	0.5	1	8	0.5
577128	SAMEA5608809	0.004	<0.002	<0.016	0.5	0.125	8	0.5
577223	SAMEA5608810	0.004	0.002	<0.016	0.5	0.125	16	1
577226	SAMEA5608811	2	0.004	<0.016	0.5	0.25	16	1
577230	SAMEA5608812	8	0.032	0.032	0.5	0.5	16	1
577530	SAMEA5608813	0.004	<0.002	<0.016	0.064	0.064	8	0.125
577534	SAMEA5608814	0.004	0.002	<0.016	0.5	0.125	16	0.5
577537	SAMEA5608815	4	0.008	<0.016	0.25	0.5	16	2
577543	SAMEA5608816	2	0.002	<0.016	0.125	8	8	16
577547	SAMEA5608817	16	0.008	<0.016	2	0.5	16	1
577551	SAMEA5608818	0.004	0.002	<0.016	0.5	0.25	16	0.5
577555	SAMEA5608819	0.004	0.002	<0.016	0.5	0.25	16	1
577558	SAMEA5608820	0.004	0.002	<0.016	0.25	0.125	16	0.5
577666	SAMEA5608821	0.002	0.004	<0.016	0.064	0.25	16	0.125
577883	SAMEA5608822	0.004	0.002	<0.016	0.5	0.125	16	1

577889	SAMEA5608823	0.004	0.002	<0.016	0.25	0.125	16	0.5
577910	SAMEA5608824	0.004	0.004	<0.016	0.125	0.125	16	0.125
577912	SAMEA5608825	0.004	0.002	<0.016	0.25	0.125	8	0.25
577914	SAMEA5608826	0.008	0.002	<0.016	0.5	0.125	16	0.25
577916	SAMEA5608827	0.004	0.002	<0.016	1	0.125	16	1
577982	SAMEA5608828	1	0.008	<0.016	0.125	>32	16	16
578271	SAMEA5608829	2	0.004	<0.016	0.5	>32	16	0.5
578273	SAMEA5608830	0.004	0.004	<0.016	0.125	0.064	16	0.25
578289	SAMEA5608831	0.002	<0.002	<0.016	0.25	0.25	16	0.25
578291	SAMEA5608832	4	0.016	0.032	0.25	0.25	8	0.5
578293	SAMEA5608833	0.004	0.002	<0.016	0.125	0.125	16	32
578295	SAMEA5608834	0.002	<0.002	<0.016	1	0.125	16	0.5
578297	SAMEA5608835	0.004	0.002	<0.016	0.25	0.125	16	0.5
578299	SAMEA5608836	0.004	<0.002	<0.016	0.064	0.125	16	16
578697	SAMEA5608837	0.004	<0.002	<0.016	0.5	0.125	16	0.5
578708	SAMEA5608838	0.004	0.002	<0.016	0.25	2	16	0.5
578712	SAMEA5608839	4	0.016	0.032	0.25	0.25	8	0.5
579028	SAMEA5608840	0.002	0.002	<0.016	0.5	0.125	16	0.5
579030	SAMEA5608841	0.002	0.002	<0.016	0.5	0.125	16	0.5
579032	SAMEA5608842	0.004	0.004	<0.016	0.25	0.25	16	0.5
579034	SAMEA5608843	0.004	0.002	<0.016	0.5	0.25	16	0.5
579036	SAMEA5608844	4	0.004	<0.016	0.25	0.25	16	32
579116	SAMEA5608845	>32	0.016	<0.016	0.5	2	16	2
579358	SAMEA5608846	4	0.008	<0.016	0.5	0.5	16	0.5
579421	SAMEA5608847	0.004	0.004	<0.016	0.5	0.25	16	0.125
579424	SAMEA5608848	0.004	0.004	<0.016	0.125	0.5	16	0.25
580143	SAMEA5608849	0.004	<0.002	<0.016	0.064	0.125	16	16
580145	SAMEA5608850	0.004	0.004	<0.016	0.25	0.25	16	1
580147	SAMEA5608851	0.004	0.004	<0.016	0.25	0.25	16	1
580400	SAMEA5608852	0.004	0.004	<0.016	0.5	0.125	16	0.5
580422	SAMEA5608853	0.004	0.002	<0.016	0.5	0.25	16	0.25
580429	SAMEA5608854	4	0.008	0.032	0.064	0.25	16	0.5
580458	SAMEA5608855	>32	0.125	0.125	0.5	2	16	2
580466	SAMEA5608856	0.004	0.002	<0.016	0.5	0.25	16	0.25
580468	SAMEA5608857	0.004	0.002	<0.016	0.125	0.25	16	16
580470	SAMEA5608858	2	0.008	<0.016	0.25	0.5	16	0.5

580683	SAMEA5608859	0.004	0.008	0.016	0.125	0.25	16	1
580685	SAMEA5608860	8	0.016	0.016	0.125	8	16	1
580687	SAMEA5608861	0.004	<0.002	<0.016	0.5	0.25	8	0.5
580689	SAMEA5608862	0.004	0.004	<0.016	0.125	0.25	16	0.25
581036	SAMEA5608863	16	0.125	0.064	0.25	2	16	2
589854	SAMEA5608864	4	0.004	<0.016	0.064	2	16	32
590003	SAMEA5608865	0.004	<0.002	<0.016	0.25	0.125	16	0.25
590005	SAMEA5608866	0.008	0.004	<0.016	0.25	0.25	16	0.25
590200	SAMEA5608867	0.004	<0.002	<0.016	0.25	0.016	16	0.125
590462	SAMEA5608868	0.008	0.004	<0.016	0.25	0.125	16	0.5
590783	SAMEA5608869	0.004	0.004	<0.016	0.5	0.25	16	0.25
590785	SAMEA5608870	4	0.008	<0.016	0.25	1	8	0.5
590788	SAMEA5608871	2	0.016	0.032	0.5	0.5	16	1
590792	SAMEA5608872	4	0.008	0.016	0.5	0.5	16	1
590794	SAMEA5608873	0.016	0.008	<0.016	2	0.5	16	1
590801	SAMEA5608874	0.004	<0.002	<0.016	0.5	0.25	16	0.5
590884	SAMEA5608875	0.004	0.004	<0.016	0.25	0.25	16	0.25
590887	SAMEA5608876	4	0.016	0.064	0.5	0.5	16	1
591244	SAMEA5608877	2	0.008	<0.016	0.125	16	8	64
591246	SAMEA5608878	0.002	<0.002	<0.016	0.25	0.125	8	0.25
591253	SAMEA5608879	2	0.004	<0.016	0.25	>32	16	0.5
591259	SAMEA5608880	0.004	0.002	<0.016	0.125	0.125	8	32
591415	SAMEA5608881	16	0.032	0.032	0.5	0.5	16	1
591417	SAMEA5608882	0.002	0.008	0.016	0.125	0.5	8	0.5
591426	SAMEA5608883	8	0.064	0.064	0.25	2	8	2
591432	SAMEA5608884	0.004	0.004	<0.016	0.25	0.25	16	0.25
591434	SAMEA5608885	0.004	0.004	<0.016	0.25	0.125	16	0.5
591436	SAMEA5608886	0.004	0.002	<0.016	0.125	0.125	16	32
591438	SAMEA5608887	0.008	0.004	<0.016	1	0.25	8	0.5
591620	SAMEA5608888	0.004	0.002	<0.016	0.5	0.25	16	0.5
591736	SAMEA5608889	1	0.002	<0.016	0.25	>32	8	0.5
591740	SAMEA5608890	0.004	0.002	<0.016	0.5	0.25	16	0.5
591881	SAMEA5608891	8	0.004	<0.016	0.125	32	16	32
591882	SAMEA5608892	2	0.002	<0.016	0.125	32	16	32
591886	SAMEA5608893	2	0.004	<0.016	0.25	>32	16	0.5
591982	SAMEA5608894	0.004	0.004	<0.016	0.5	0.25	16	0.25

592017	SAMEA5608895	0.004	<0.002	<0.016	0.064	0.125	16	0.5
592026	SAMEA5608896	>32	0.032	0.016	0.5	2	16	4
592092	SAMEA5608897	8	0.008	<0.016	0.25	0.5	16	0.5
592330	SAMEA5608898	4	0.002	<0.016	0.125	>32	16	8
592339	SAMEA5608899	2	0.002	<0.016	0.125	0.25	8	16
592342	SAMEA5608900	0.002	<0.002	<0.016	0.125	0.016	8	0.125
592348	SAMEA5608901	0.002	<0.002	<0.016	0.125	0.016	8	0.125
592358	SAMEA5608902	0.004	0.008	<0.016	0.125	0.25	16	1
592360	SAMEA5608903	0.004	0.004	<0.016	0.5	0.25	8	0.25
592362	SAMEA5608904	0.004	0.004	<0.016	0.25	0.25	16	1
592364	SAMEA5608905	0.008	0.004	<0.016	0.25	0.25	8	0.25
592415	SAMEA5608906	0.125	0.004	<0.016	0.064	2	16	1
592483	SAMEA5608907	0.004	0.004	<0.016	0.25	0.25	16	1
592485	SAMEA5608908	0.004	0.002	<0.016	0.5	0.25	16	0.25
592487	SAMEA5608909	0.004	0.004	<0.016	0.5	0.25	16	0.5
592489	SAMEA5608910	4	0.002	<0.016	16	0.5	8	1
592584	SAMEA5608911	1	0.002	<0.016	0.125	0.25	16	16
592586	SAMEA5608912	>32	0.032	0.032	0.25	0.5	8	1
592616	SAMEA5608913	2	0.008	<0.016	0.25	4	8	1
592618	SAMEA5608914	8	0.008	<0.016	0.25	0.5	8	1
592620	SAMEA5608915	0.002	0.002	<0.016	0.5	0.125	8	0.5
592622	SAMEA5608916	0.004	0.002	<0.016	0.5	0.25	8	0.5
592624	SAMEA5608917	0.004	0.002	<0.016	0.5	0.25	16	0.5
592626	SAMEA5608919	0.004	<0.002	<0.016	0.5	0.25	8	0.125
592628	SAMEA5608920	0.004	0.002	<0.016	0.5	0.125	16	1
592633	SAMEA5608921	0.004	0.002	<0.016	0.5	0.25	8	0.5
592686	SAMEA5608922	2	0.004	<0.016	0.5	>32	16	0.5
592898	SAMEA5608923	0.004	0.002	<0.016	0.125	0.125	16	0.5
592900	SAMEA5608924	0.004	0.002	<0.016	0.5	0.125	16	1
592902	SAMEA5608925	>32	0.064	0.064	0.25	1	8	2
592904	SAMEA5608926	0.004	0.002	<0.016	0.25	0.25	16	0.25
592911	SAMEA5608927	0.004	0.002	<0.016	0.25	0.25	8	0.25
592913	SAMEA5608928	16	0.064	0.064	0.25	1	16	2
592915	SAMEA5608929	0.004	0.004	<0.016	0.5	0.25	16	0.5
592922	SAMEA5608930	0.002	<0.002	<0.016	0.064	0.008	8	0.125
592924	SAMEA5608931	0.004	0.002	<0.016	0.5	0.25	16	0.25

593063	SAMEA5608932	0.004	0.002	<0.016	0.5	0.25	16	0.125
593076	SAMEA5608933	0.002	<0.002	<0.016	0.064	0.008	16	0.125
593227	SAMEA5608934	0.004	0.002	<0.016	0.5	0.25	16	0.125
593229	SAMEA5608935	0.004	0.002	<0.016	0.25	0.25	8	0.25
593231	SAMEA5608936	0.004	0.002	<0.016	0.25	0.25	8	0.25
593233	SAMEA5608937	0.004	0.002	<0.016	0.25	0.25	8	0.25
593235	SAMEA5608938	0.004	0.002	<0.016	0.5	0.125	16	1
593237	SAMEA5608939	0.004	<0.002	<0.016	0.25	0.25	8	0.25
593239	SAMEA5608940	0.004	<0.002	<0.016	0.5	0.25	16	0.25
593241	SAMEA5608941	0.004	0.004	<0.016	0.5	0.25	16	0.25
593243	SAMEA5608942	0.004	0.002	<0.016	0.125	0.125	8	0.25
593245	SAMEA5608943	0.004	0.004	<0.016	0.25	0.25	16	1
593247	SAMEA5608944	1	0.004	<0.016	0.125	0.25	16	32
593250	SAMEA5608945	0.004	<0.002	<0.016	0.25	0.25	16	0.25
593252	SAMEA5608946	0.004	0.002	<0.016	0.25	0.125	16	1
593683	SAMEA5608947	0.016	0.004	<0.016	0.25	4	16	1
593686	SAMEA5608948	4	0.004	<0.016	0.5	0.5	8	0.5
593688	SAMEA5608949	2	0.008	0.016	0.25	0.5	16	32
593763	SAMEA5608950	0.004	0.002	<0.016	0.5	0.25	8	0.25
593768	SAMEA5608951	4	0.004	<0.016	0.25	8	8	64
593771	SAMEA5608952	0.002	0.002	<0.016	4	0.5	16	1
593781	SAMEA5608953	0.002	<0.002	<0.016	0.5	2	8	0.5
593785	SAMEA5608954	0.004	0.004	<0.016	0.5	0.064	8	1
593788	SAMEA5608955	0.004	0.002	<0.016	0.25	0.125	16	1
593792	SAMEA5608956	2	0.008	0.032	0.032	0.25	8	0.5
593794	SAMEA5608957	0.002	0.002	<0.016	0.25	0.25	8	0.5
593927	SAMEA5608958	8	0.002	<0.016	0.25	0.25	16	1
593973	SAMEA5608959	0.002	0.002	<0.016	0.5	0.125	16	0.5
594101	SAMEA5608960	0.004	0.002	<0.016	0.125	0.125	8	64
594113	SAMEA5608961	2	0.004	<0.016	0.125	16	16	32
594119	SAMEA5608962	2	0.002	<0.016	0.064	16	8	32
594125	SAMEA5608963	0.004	0.002	<0.016	0.125	0.125	8	0.25
594132	SAMEA5608964	16	0.004	<0.016	0.5	1	16	1
594135	SAMEA5608965	0.004	0.002	<0.016	0.5	0.125	8	0.25
594138	SAMEA5608966	0.004	0.002	<0.016	0.5	0.125	16	1
594278	SAMEA5608967	2	0.004	<0.016	0.125	2	4	32

594280	SAMEA5608968	0.004	0.002	<0.016	0.25	0.125	16	0.5
594354	SAMEA5608969	0.004	0.002	<0.016	0.5	0.125	8	0.5
594361	SAMEA5608970	0.004	0.002	<0.016	0.125	0.064	8	16
594368	SAMEA5608971	2	0.004	<0.016	0.125	0.5	8	0.5
594376	SAMEA5608972	0.002	0.002	<0.016	2	0.125	16	1
594454	SAMEA5608973	2	0.002	<0.016	0.125	0.125	8	16
594498	SAMEA5608974	0.002	0.002	<0.016	0.5	0.064	8	0.5
594502	SAMEA5608975	4	0.002	<0.016	0.25	0.125	8	0.5
594673	SAMEA5608976	2	0.008	<0.016	0.125	4	8	16
594675	SAMEA5608977	0.002	0.002	<0.016	0.5	0.064	16	0.5
594677	SAMEA5608978	0.004	0.004	<0.016	0.5	0.125	8	0.25
594679	SAMEA5608979	2	0.004	<0.016	0.125	4	16	16
594681	SAMEA5608980	0.004	<0.002	<0.016	0.125	0.016	16	0.25
594683	SAMEA5608981	4	0.002	<0.016	0.125	8	8	16
594745	SAMEA5608982	0.008	0.004	<0.016	0.25	0.125	8	0.25
594881	SAMEA5608983	>32	0.064	0.064	0.25	1	8	2
594887	SAMEA5608984	0.004	0.002	<0.016	0.5	0.125	16	0.25
595329	SAMEA5608985	8	0.064	0.064	0.5	1	16	2
595332	SAMEA5608986	2	0.004	<0.016	0.25	>32	16	32
595336	SAMEA5608987	0.004	0.004	<0.016	0.5	0.25	8	0.25
595600	SAMEA5608988	2	0.008	<0.016	0.25	2	4	16
595638	SAMEA5608989	0.004	0.004	<0.016	0.5	0.125	16	0.5
595640	SAMEA5608990	0.004	0.008	0.016	0.5	0.5	16	1
595643	SAMEA5608991	8	0.002	<0.016	0.125	0.032	0.5	0.5
595645	SAMEA5608992	0.004	0.002	<0.016	0.5	0.125	8	0.25
595647	SAMEA5608993	0.004	0.002	<0.016	0.25	0.125	16	1
595710	SAMEA5608994	8	0.004	<0.016	0.5	0.25	16	1
595712	SAMEA5608995	0.004	0.004	<0.016	0.5	0.25	16	0.25
595714	SAMEA5608996	>32	0.016	0.016	0.5	1	16	2
595716	SAMEA5608997	8	0.016	0.032	0.125	32	8	0.5
595720	SAMEA5608998	4	0.004	<0.016	0.5	0.25	8	0.5
595722	SAMEA5608999	4	0.008	0.016	0.125	2	8	32
595726	SAMEA5609000	0.004	0.002	<0.016	0.5	0.125	8	0.25
595728	SAMEA5609001	>32	0.008	<0.016	2	0.25	16	2
595943	SAMEA5609002	2	0.004	<0.016	1	0.125	8	1
595945	SAMEA5609003	8	0.004	<0.016	0.125	2	16	16

595947	SAMEA5609004	0.004	0.002	<0.016	0.5	0.125	16	0.5
596208	SAMEA5609005	0.004	<0.002	<0.016	0.5	0.125	8	0.25
596213	SAMEA5609006	0.002	<0.002	<0.016	0.5	0.064	16	0.5
596216	SAMEA5609007	2	0.004	<0.016	0.25	0.25	8	0.5
596218	SAMEA5609008	0.004	0.002	<0.016	0.5	0.125	16	0.5
596220	SAMEA5609009	>32	0.064	0.064	0.25	1	8	2
596222	SAMEA5609010	0.004	0.002	<0.016	0.125	0.125	8	16
596307	SAMEA5609011	0.004	0.004	<0.016	0.5	0.064	8	0.25
596321	SAMEA5609012	0.004	0.004	<0.016	0.25	0.125	8	1
596543	SAMEA5609013	0.002	0.002	<0.016	0.032	4	8	0.25
596545	SAMEA5609014	0.004	0.002	<0.016	0.5	0.125	16	0.5
596547	SAMEA5609015	0.004	0.002	<0.016	0.5	0.125	16	0.5
596549	SAMEA5609016	4	0.008	<0.016	0.5	0.5	16	1
596551	SAMEA5609017	16	0.004	<0.016	0.25	8	16	1
596654	SAMEA5609018	0.004	0.004	0.016	0.25	0.25	16	0.25
596761	SAMEA5609019	0.004	0.002	<0.016	0.125	0.125	16	0.5
596907	SAMEA5609020	0.004	0.004	<0.016	0.25	0.125	8	0.25
596916	SAMEA5609021	2	0.004	<0.016	0.125	0.25	16	16
596947	SAMEA5609022	0.004	0.002	<0.016	0.5	0.125	8	0.25
597251	SAMEA5609023	8	0.008	<0.016	0.25	0.25	8	0.5
597254	SAMEA5609024	0.008	0.002	<0.016	0.25	0.125	16	0.25
597257	SAMEA5609025	0.004	0.002	<0.016	0.5	0.125	8	0.5
597321	SAMEA5609026	4	0.008	0.064	0.125	0.125	16	1
597323	SAMEA5609027	0.004	0.002	<0.016	0.125	0.064	8	16
597325	SAMEA5609028	0.004	0.004	<0.016	0.5	0.25	8	0.25
597327	SAMEA5609029	2	0.002	<0.016	<0.016	0.125	8	16
597527	SAMEA5609030	4	0.004	<0.016	0.125	16	16	32
597645	SAMEA5609031	4	0.016	0.032	0.125	0.25	8	0.25
597648	SAMEA5609032	0.008	0.004	<0.016	0.125	0.125	16	32
597650	SAMEA5609033	>32	0.125	0.125	0.5	2	8	2
597652	SAMEA5609034	2	0.002	<0.016	0.064	>32	8	8
598028	SAMEA5609035	>32	0.064	0.064	0.25	1	16	2
598030	SAMEA5609036	1	0.004	<0.016	0.125	0.25	16	32
598032	SAMEA5609037	0.004	0.002	<0.016	0.5	0.25	16	0.25
598034	SAMEA5609038	0.004	0.002	<0.016	0.5	0.125	16	1
598207	SAMEA5609039	0.008	0.002	<0.016	0.5	0.125	16	0.25

598234	SAMEA5609040	0.004	0.002	<0.016	0.25	0.125	16	32
598238	SAMEA5609041	8	0.008	<0.016	1	0.5	16	1
598593	SAMEA5609042	2	0.004	<0.016	0.032	>32	8	16
598596	SAMEA5609043	2	0.002	<0.016	0.064	>32	8	16
598643	SAMEA5609044	2	0.004	<0.016	0.5	>32	16	1
598782	SAMEA5609045	16	0.064	0.064	0.25	1	8	2
598784	SAMEA5609046	16	0.125	0.125	0.25	1	8	2
598786	SAMEA5609047	0.004	0.002	<0.016	0.5	0.064	8	0.5
598788	SAMEA5609048	8	0.004	<0.016	0.25	0.25	8	0.5
598807	SAMEA5609049	0.004	0.002	<0.016	0.5	0.25	16	0.25
599026	SAMEA5609050	0.004	0.032	0.032	0.5	0.5	16	1
599029	SAMEA5609051	0.004	0.004	<0.016	0.5	0.25	8	0.25
599339	SAMEA5609052	0.008	0.004	<0.016	0.5	0.25	16	0.25
599341	SAMEA5609053	0.008	0.004	<0.016	0.5	0.25	16	0.25
599343	SAMEA5609054	0.004	0.002	<0.016	0.125	0.25	8	1
599345	SAMEA5609055	0.004	0.004	<0.016	0.5	0.25	16	0.5
599347	SAMEA5609056	>32	0.125	0.125	0.25	2	16	2
599349	SAMEA5609057	0.004	0.004	<0.016	0.5	0.25	16	0.25
599618	SAMEA5609058	0.008	0.004	<0.016	0.25	0.125	16	0.25
599759	SAMEA5609059	0.032	0.004	<0.016	0.064	>32	8	128
599763	SAMEA5609060	0.016	0.016	0.032	1	1	16	2
599767	SAMEA5609061	2	0.016	0.016	0.064	8	8	32
599991	SAMEA5609062	0.002	0.002	<0.016	0.25	0.064	8	0.5
599997	SAMEA5609063	0.008	0.004	<0.016	0.125	0.125	8	32
600006	SAMEA5609064	0.002	0.002	<0.016	0.125	0.125	8	0.5
600009	SAMEA5609065	0.002	0.002	<0.016	0.25	0.064	8	0.25
600013	SAMEA5609066	>32	0.016	0.032	0.25	0.25	16	1
600016	SAMEA5609067	0.004	0.002	<0.016	0.25	0.125	16	0.25
600020	SAMEA5609068	32	0.064	0.064	0.25	1	8	2
600022	SAMEA5609069	>32	0.125	0.125	0.25	1	16	2
600025	SAMEA5609070	0.008	0.002	<0.016	0.5	0.25	16	0.5
600027	SAMEA5609071	0.004	0.002	<0.016	0.125	0.125	8	32
600089	SAMEA5609072	0.004	0.002	<0.016	0.25	0.064	16	1
600167	SAMEA5609073	0.004	0.004	<0.016	0.25	0.25	16	1
600294	SAMEA5609074	0.008	0.002	<0.016	0.25	0.125	16	1
600507	SAMEA5609075	2	0.004	<0.016	0.064	0.25	16	1

600737	SAMEA5609076	2	0.004	<0.016	1	0.25	16	1
600743	SAMEA5609077	0.004	0.002	<0.016	0.5	0.125	16	1
600751	SAMEA5609078	16	0.004	<0.016	0.5	1	8	1
600792	SAMEA5609079	0.002	0.002	<0.016	1	0.25	8	0.5
601023	SAMEA5609080	>32	0.016	0.064	1	0.5	8	4
601031	SAMEA5609081	8	0.004	<0.016	1	0.125	16	32
601069	SAMEA5609082	>32	0.125	0.125	0.25	2	16	4
601071	SAMEA5609083	4	0.004	<0.016	0.125	>32	8	16
601074	SAMEA5609084	0.004	0.002	<0.016	0.5	0.125	8	0.5
601197	SAMEA5609085	0.004	0.004	<0.016	0.125	0.064	8	0.25
601476	SAMEA5609086	>32	0.016	0.016	0.5	1	8	64
601479	SAMEA5609087	0.008	0.004	<0.016	0.25	0.25	16	1
601487	SAMEA5609088	0.004	0.004	<0.016	1	0.25	16	0.25
601489	SAMEA5609089	4	0.004	<0.016	0.25	1	16	0.5
601684	SAMEA5609090	0.008	0.004	<0.016	0.25	0.25	16	0.5
601691	SAMEA5609091	4	0.008	0.064	0.064	0.25	16	0.5
601695	SAMEA5609092	0.004	0.004	<0.016	1	0.125	16	1
601701	SAMEA5609093	4	0.016	0.064	0.064	0.25	16	0.5
601703	SAMEA5609094	2	0.008	0.032	0.064	0.125	16	0.5
601767	SAMEA5609095	2	0.004	<0.016	0.064	8	16	8
601806	SAMEA5609096	2	0.004	<0.016	0.064	8	8	8
601812	SAMEA5609097	0.004	0.002	<0.016	0.5	0.25	16	0.25
601853	SAMEA5609098	1	0.004	<0.016	0.125	4	16	16
601955	SAMEA5609099	0.004	<0.002	<0.016	0.5	0.125	8	0.25
601961	SAMEA5609100	0.004	0.002	<0.016	0.25	0.125	8	0.25
601963	SAMEA5609101	1	0.008	<0.016	0.125	8	16	16
602142	SAMEA5609102	0.004	0.004	<0.016	0.5	0.125	16	0.5
602144	SAMEA5609103	2	0.032	0.032	0.25	0.5	16	1
602285	SAMEA5609104	0.016	0.004	<0.016	0.25	0.25	16	0.5
602453	SAMEA5609105	>32	0.032	0.064	0.5	1	16	2
602509	SAMEA5609106	0.008	0.004	<0.016	0.5	0.25	16	0.25
602512	SAMEA5609107	16	0.008	0.064	0.25	0.25	8	0.5
602514	SAMEA5609108	1	0.004	<0.016	0.125	2	16	16
603155	SAMEA5609109	0.004	0.002	<0.016	0.25	0.125	8	0.5
603166	SAMEA5609110	0.004	0.004	<0.016	0.5	0.25	16	0.5
603169	SAMEA5609111	0.004	0.004	<0.016	0.25	0.25	16	32

603171	SAMEA5609112	0.004	0.002	<0.016	0.25	0.125	16	0.25
603175	SAMEA5609113	0.004	0.002	<0.016	0.125	0.064	16	32
603264	SAMEA5609114	1	0.004	<0.016	0.125	4	16	32
603435	SAMEA5609115	0.004	0.002	<0.016	0.5	0.125	8	0.25
603442	SAMEA5609116	0.004	0.002	<0.016	0.5	0.25	16	0.5
603446	SAMEA5609117	0.004	0.002	<0.016	0.25	0.125	16	0.5
603449	SAMEA5609118	0.032	0.004	<0.016	>256	0.5	16	1
603452	SAMEA5609119	16	0.032	0.032	0.5	0.5	8	1
603457	SAMEA5609120	0.032	0.004	<0.016	>256	0.5	16	1
603610	SAMEA5609121	0.004	0.004	<0.016	0.5	0.25	16	0.25
603813	SAMEA5609122	2	0.002	<0.016	0.125	32	16	0.25
603932	SAMEA5609123	4	0.004	<0.016	0.5	0.25	16	32
603939	SAMEA5609124	8	0.032	0.032	0.25	0.5	16	1
603942	SAMEA5609125	2	0.002	<0.016	0.25	>32	16	0.25
604158	SAMEA5609126	0.004	<0.002	<0.016	0.5	0.125	16	0.25
604161	SAMEA5609127	0.004	0.002	<0.016	0.5	0.125	16	0.5
604965	SAMEA5609128	0.016	0.004	<0.016	>256	0.5	16	2
604973	SAMEA5609129	0.016	0.008	<0.016	2	0.5	16	1
604993	SAMEA5609130	32	0.008	<0.016	2	0.5	16	2
605007	SAMEA5609131	16	0.008	<0.016	2	0.5	16	2
605009	SAMEA5609132	0.004	0.004	<0.016	0.25	0.25	16	1
605153	SAMEA5609133	16	0.008	<0.016	2	0.5	16	2
605202	SAMEA5609134	0.004	0.004	<0.016	0.25	0.25	16	1
605204	SAMEA5609135	0.002	0.002	<0.016	0.125	0.125	8	32
605206	SAMEA5609136	2	0.002	<0.016	0.5	0.25	16	0.5
605533	SAMEA5609137	0.004	<0.002	<0.016	0.125	0.064	16	0.5
605694	SAMEA5609138	16	0.064	0.064	0.25	1	8	1
605702	SAMEA5609139	16	0.064	0.064	0.25	1	16	2
605704	SAMEA5609140	0.004	0.002	<0.016	0.25	0.064	16	0.5
605723	SAMEA5609141	8	0.008	0.016	2	0.25	16	2
605764	SAMEA5609142	2	0.016	0.032	0.25	0.5	16	0.5
605803	SAMEA5609143	2	0.004	<0.016	0.125	0.25	8	32
605952	SAMEA5609144	1	0.004	<0.016	0.064	0.25	8	16
605955	SAMEA5609145	4	0.008	<0.016	0.25	0.5	16	0.5
607589	SAMEA5609146	0.004	<0.002	<0.016	0.25	0.032	16	0.25
607789	SAMEA5609147	16	0.008	<0.016	2	0.5	16	1

608526	SAMEA5609148	8	0.008	<0.016	2	0.5	16	2
609222	SAMEA5609149	1	0.008	<0.016	0.125	2	8	32
609602	SAMEA5609150	8	0.004	<0.016	0.125	>32	8	32
609610	SAMEA5609151	0.008	0.002	<0.016	0.5	0.25	16	0.5
609666	SAMEA5609152	16	0.064	0.064	0.25	1	8	2
610072	SAMEA5609153	8	0.008	0.032	1	0.25	8	1
610074	SAMEA5609154	0.004	0.004	<0.016	0.125	0.125	16	1
610096	SAMEA5609155	0.004	0.002	<0.016	0.25	0.25	8	0.25
610098	SAMEA5609156	0.004	0.002	<0.016	0.25	0.125	16	0.5
610100	SAMEA5609157	0.004	0.002	<0.016	0.5	0.25	8	0.25
610102	SAMEA5609158	4	0.002	<0.016	0.5	0.25	16	0.5
610104	SAMEA5609159	4	0.002	<0.016	0.5	0.25	16	0.5
610106	SAMEA5609160	0.002	<0.002	<0.016	0.032	0.064	8	0.125
610110	SAMEA5609161	16	0.032	0.064	0.5	0.5	16	1
610112	SAMEA5609162	32	0.032	0.032	0.5	0.5	16	1
610219	SAMEA5609163	16	0.008	0.064	0.125	0.25	8	0.5
610347	SAMEA5609164	0.004	0.002	<0.016	0.25	0.25	8	0.25
610559	SAMEA5609165	16	0.008	<0.016	4	0.5	16	2
610601	SAMEA5609166	>32	0.016	0.064	1	2	16	2
610748	SAMEA5609167	4	0.002	<0.016	0.064	8	16	16
610754	SAMEA5609168	16	0.008	<0.016	2	0.5	16	1
610759	SAMEA5609169	8	0.004	<0.016	0.5	>32	16	1
610780	SAMEA5609170	16	0.008	<0.016	2	0.5	16	2
610786	SAMEA5609171	0.004	0.004	<0.016	0.25	0.125	16	1
